# Clove Essential Oil as an Antifungal Agent and Putative Dual-Action Antifungal Mechanism: Experimental Validation and Computational Insights into Orthosteric and Allosteric Modulation of Chitin Synthase I in *Penicillium* Species

**DOI:** 10.3390/molecules31071132

**Published:** 2026-03-30

**Authors:** Yamid Castillo, Edgar A. Márquez Brazón, Yeimmy Peralta-Ruiz, Clemencia Chaves-López, Oscar Saurith-Coronell, Carlos David Grande-Tovar

**Affiliations:** 1 Grupo de Investigación de Fotoquímica y Fotobiología, Universidad del Atlántico, Carrera 30 Número 8-49, Puerto Colombia 081008, Colombia; yacastillo@mail.uniatlantico.edu.co; 2 Departamento de Química y Biología, Facultad de Ciencias Básicas, Universidad del Norte, Barranquilla 080020, Colombia; ebrazon@uninorte.edu.co (E.A.M.B.); osaurith@uninorte.edu.co (O.S.-C.); 3 Programa de Ingeniería Agroindustrial, Facultad de Ingeniería, Universidad del Atlántico, Carrera 30 Número 8-49, Puerto Colombia 081008, Colombia; yeimmyperalta@mail.uniatlantico.edu.co; 4 Department of Bioscience and Technology for Food, Agriculture and Environment, University of Teramo, Via R. Balzarini 1, 64100 Teramo, Italy; cchaveslopez@unite.it; 5 Departamento de Medicina, División Ciencias de la Salud, Universidad del Norte, Km 5, Vía Puerto Colombia, Puerto Colombia 081007, Colombia

**Keywords:** clove essential oil, *P. expansum*, *P. brevicompactum*, chitin synthase I (CHS I), natural antifungals, allosteric inhibition

## Abstract

Fungal contamination during postharvest storage causes significant food losses, particularly due to *Penicillium expansum* and *Penicillium brevicompactum*, highlighting the need for sustainable antifungal alternatives. This study evaluated the antifungal potential of clove essential oil (*Syzygium aromaticum*) against *P. expansum* and *P. brevicompactum* by integrating in vitro assays with in silico analyses. Minimum inhibitory concentrations (MICs) were determined, and effects on fungal growth, membrane integrity, and spore germination were assessed. Molecular docking and molecular dynamics simulations were performed to evaluate the affinity and stability of the five most abundant GC–MS compounds that met predefined ProTox-II toxicity criteria (categories 5–6; LD_50_ ≥ 2000 mg/kg) toward chitin synthase I (CHS I), a key enzyme in chitin biosynthesis. The oil exhibited strong inhibitory activity, with MIC values of 0.156 µL/mL against *P. expansum* and 0.312 µL/mL against *P. brevicompactum*, along with significant morphological and physiological alterations. Computational analyses indicated that trans-β-caryophyllene oxide and α-humulene form stable interactions at both the active and an allosteric site of CHS I, supporting a putative dual inhibitory mechanism. These findings highlight clove essential oil as a promising ecological alternative to synthetic fungicides and underscore the value of computational approaches for elucidating antifungal mechanisms in understudied species.

## 1. Introduction

Postharvest fungal contamination is a major contributor to global food loss, threatening food security and sustainability. The Food and Agriculture Organization (FAO) estimates that approximately 1.3 billion tons of food are wasted annually, with fruits and vegetables among the most affected commodities. In Latin America, postharvest losses reach nearly 34% of total production, significantly impacting economic sustainability and contributing to greenhouse gas emissions [1].

Among the leading causes of post-harvest deterioration are fungal infections. For instance, *Penicillium brevicompactum* affects some fruits such as grapes and dairy products (yogurt) [2,3], and is known to produce mycotoxins such as mycophenolic acid during storage, which compromises food safety and quality [2,4], while *Penicillium expansum* produces the characteristic blue mold responsible for losses in fruits such as apples, pears, cherries, and is an important producer of mycotoxins such as patulin [5,6]. Although synthetic pesticides are the most widely used control strategy, their use causes physicochemical and biological alterations in the soil, oxidative stress in plants, and adverse effects on human health, including exacerbation of respiratory diseases, genetic alterations, and potential carcinogenicity. These limitations have prompted the search for more sustainable alternatives [7,8].

Among the emerging options, essential oils (EOs) stand out for their rich terpene, terpenoid, and aromatic compound composition, as well as their well-established antifungal activity and antioxidant capacity. Several essential oils have demonstrated activity against postharvest pathogens, particularly those rich in phenolic compounds such as thymol, carvacrol, and cinnamaldehyde, among others, which can disrupt fungal membranes and interfere with cellular metabolism. Oils obtained from species such as thyme, oregano, cinnamon, and related plants have been extensively researched for the control of postharvest diseases in fruits [9,10]. However, the practical application of some essential oils in food systems can be limited by their strong sensory characteristics and the relatively high concentrations required to achieve antimicrobial efficacy [10]. In contrast, clove essential oil has attracted increasing attention due to its high eugenol content and compatibility with food applications, where it has been incorporated into edible coatings and preservation strategies to extend the shelf life of fruits and other products [11,12]. Furthermore, Clove essential oil (CEO) has shown significant efficacy against phytopathogenic fungi, including *Aspergillus* spp. and *Trichothecium* spp. Its potential also extends to applications such as coatings for fruits and vegetables, helping to extend the shelf life of agricultural products [13,14]. However, it is necessary to identify effective concentrations and determine which specific components are responsible for the observed antifungal activity.

Several studies have demonstrated the efficacy of essential oils as postharvest antifungal treatments against *Penicillium* species responsible for fruit decay. For example, oregano essential oil has been reported to significantly reduce blue mold caused by *P. expansum* in stored fruit [15,16]. In addition, vapor phase applications have been shown to have inhibitory effects against *Penicillium* spp. after harvest under storage conditions [17,18]. Despite these promising results, the practical application of essential oils remains limited by their high volatility, low water solubility, and stability issues in storage and packaging systems [19,20]. These limitations highlight the need for a more thorough understanding of the mechanism of their antifungal activity.

Despite the biological and agricultural relevance of *P. expansum* and *P. brevicompactum*, in silico studies aimed at understanding their molecular targets remain limited. At the same time, computational approaches have been extensively applied to clinical fungi such as *Candida* and *Aspergillus* [21,22], while phytopathogenic fungi have received significantly less attention. This limits the availability of structural models, functional predictions, and ligand–protein interaction assessments that would allow for an understanding of inhibition mechanisms at the atomic level. In this context, the application of approaches such as molecular docking and molecular dynamics (MD) represents a valuable contribution to expanding knowledge about essential enzyme targets and supporting the rational design of antifungal alternatives based on naturally occurring compounds. In support of this rationale, recent case studies show how structure-guided workflows can accelerate hypothesis generation for phytopathogens and guide targeted wet-lab follow-up. For instance, in *Penicillium italicum*, Yuan et al. built a homology model of PiCYP51A (templated on human CYP51A) to perform pharmacophore-based virtual screening and docking, then prioritized hits that matched experimental EC_50_ trends, illustrating how in silico screening can bridge the gap when species-specific crystal structures are unavailable [23]. Likewise, for postharvest pathogens including *P. expansum*, Ou-Ani et al. combined GC–MS profiling with docking and 50 ns MD against a sterol demethylase target to nominate essential oil constituents for testing, exemplifying how computational readouts help deconvolve mixture effects observed in vitro [24]. More broadly, a recent review of fungal chitin synthases (CHS) highlights how emerging cryo-EM structures and comparative models now enable site-level mechanistic hypotheses and inhibitor design across fungi, precisely the kind of insight our docking/MD seeks to provide here for CHS I in *Penicillium* [25]. In this context, our computational analyses are not offered as a replacement for experiments but as a mechanism aware complement that (i) identifies orthosteric and putative allosteric regions worth probing, (ii) rationalizes why minor hydrophobic constituents may engage CHS I while eugenol chiefly contributes at the membrane level, and (iii) prioritizes residues and ligands for subsequent validation in *Penicillium* focused assays.

In silico tools, such as molecular docking and molecular dynamics, allow the prediction of covalent and non-covalent interactions between bioactive compounds and key enzymatic targets in phytopathogenic fungi [24]. One of the most critical enzyme targets in filamentous fungi is chitin synthase I (CHS I), an enzyme essential for the biosynthesis of chitin, a structural polymer composed of N-acetylglucosamine units linked by β-1,4 bonds [26]. Chitin confers rigidity, osmotic protection, and morphological support to the cell wall, in addition to performing key functions in growth, hyphal branching, and the fungus’s interaction with its environment. Chitin synthase belongs to the GT2 family of glycosyltransferases, which are responsible for catalyzing the conversion of the substrate UDP-N-acetylglucosamine (UDP-GlcNAc) into chitin chains [27]. There are seven classes of CHS grouped into two superfamilies with different catalytic activities; this diversity reflects the functional complexity of the cell wall synthesis and remodeling process [28].

Recent studies have shown that several components of essential oils, such as eugenol, humulene, and caryophyllene, have an affinity for CHS, suggesting that their antifungal activity may involve direct interference in chitin biosynthesis [29]. In this context, computational tools enable exploration of these interactions at the atomic level, predicting inhibition mechanisms and potential effects on the enzyme’s conformational stability [24].

Therefore, this study evaluated the antifungal potential of the main components of clove essential oil against *P. expansum* and *P. brevicompactum* using a combined in vitro and in silico approach. The in vitro tests enabled the determination of the essential oil’s inhibitory activity and evaluation of its effects on fungal growth, membrane integrity, and spore germination, providing direct experimental evidence of its biological efficacy. Complementarily, in silico analyses allow exploration of its affinity for CHS I and the prediction of possible mechanisms of action, providing molecular evidence supporting the use of essential oils as sustainable and safe alternatives for controlling phytopathogenic fungi. This approach contributes to the construction of ecological solutions aligned with the current demands for more sustainable agriculture.

## 2. Results and Discussion

### 2.1. In Vitro Tests

#### 2.1.1. Identification of Minimum Inhibitory Concentration (MIC), Sublethal Concentration, and Morphological Analysis

**Figure 1:**
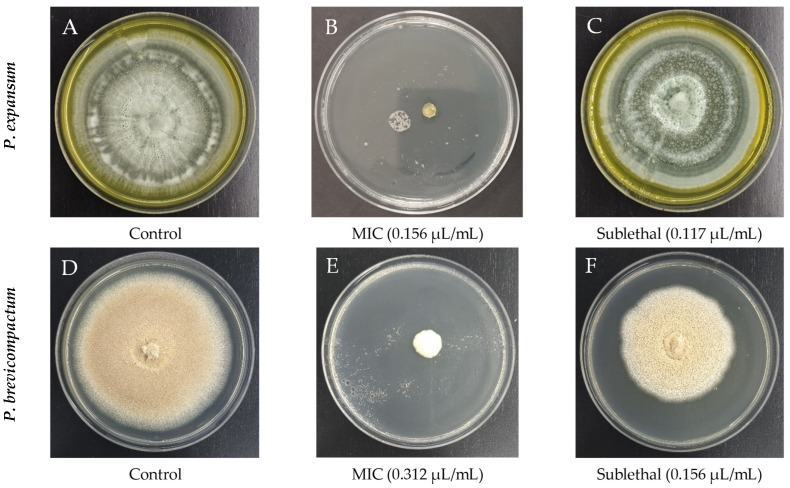
Inhibition of mycelial growth; (**A**,**D**), inoculated control on PDA agar; (**B**,**E**), MIC at a concentration of CEO; (**C**,**F**), sublethal concentration of CEO for each fungus.

After evaluating four concentrations following the methodology described in Section Effects on Mycelium Growth, the minimum inhibitory concentration (MIC) and sublethal dose were determined for both species. *P. expansum* had an MIC of 0.156 µL/mL and a sublethal dose of 0.117 µL/mL (Figure 1B,C). In *P. brevicompactum*, these values were higher, with an MIC of 0.312 µL/mL and a sublethal concentration of 0.156 µL/mL (Figure 1E,F).

At the morphological level, changes potentially associated with the essential oil were observed. In *P. expansum*, both the control and treated colonies developed concentric rings during growth; however, there were marked differences in color distribution. The control colony showed smooth transition rings in greenish-white tones (Figure 1A). In contrast, the colony exposed to the sublethal dose exhibited more defined rings, with a predominance of intense green areas and fewer whitish regions. This pattern may be related to modifications in sporulation induced by clove essential oil chemical stress [30]. In *P. brevicompactum*, the control colony grown on PDA showed typical radial growth, smooth texture, and uniform coloration characteristic of normal development (Figure 1D). In contrast, the colony exposed to the sublethal dose exhibited a more granular texture and slight color variations. These features could reflect an increase in sporulation as an adaptive response to the stress induced by the essential oil.

#### 2.1.2. Analysis of Fungal Growth Dynamics

**Figure 2:**
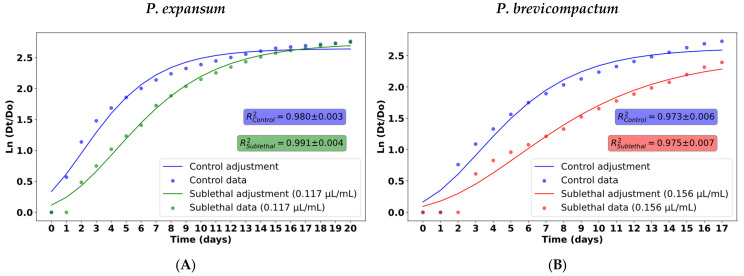
Growth dynamics for *P. expansum* (**A**) and *P. brevicompactum* (**B**) in PDA culture medium for 20 and 17 days after inoculation, respectively, at 25 °C. The blue line indicates mycelial growth control, while the green and red lines indicate mycelium treated with CEO for *P. expansum* and *P. brevicompactum*, respectively. Source: Castillo-Díaz et al., 2024 [31].

The daily mycelial growth of each species was fit to the Gompertz model (Equation (1)), yielding curves with R^2^ values between 0.97 and 0.99 (Figure 2). The parameters λ, µ_m_, and A were estimated from the model, and their values are presented in Table 1.

The λ parameter allowed us to evaluate the initial adaptation phase of each colony. In both fungi, this phase was prolonged when exposed to essential oil, suggesting metabolic stress that delayed the onset of growth. In *P. expansum*, the lag phase increased from 12 to ~36 h, while in *P. brevicompactum*, it increased from 2 to ~20 h. This delay is likely due to each species needing to adjust its enzymatic machinery and produce metabolites that counteract the effects of the essential oil [32]. The maximum growth rate (µ_m_), corresponding to the exponential phase, decreased under the sublethal concentration of essential oil in both species. Compared to the control, *P. brevicompactum* showed a 36.0 (±1.0)% reduction in µ_m_, while *P. expansum* showed a more moderate decrease of 23.9% (±5.0)%, indicating an inhibitory effect of the treatment on the growth rate [14]. Finally, parameter A, corresponding to the maximum diameter reached during the stationary phase, enabled a comparison of final growth between treatments. In *P. expansum*, the treated colony ultimately reached a final diameter slightly larger than that of the control under sublethal CEO exposure. Since the Petri dishes were sealed with parafilm throughout incubation, sustained exposure to volatile compounds was maintained, suggesting that compensatory growth in the final stage is more consistent with physiological adaptation to sublethal stress than with progressive loss of active compounds. The adaptation of fungi to antimicrobial stress has been widely documented [33,34] and may involve the activation of detoxification pathways, reinforcement of cell wall biosynthesis [35], oxidative stress responses, and metabolic reprogramming [36]. Although the mean inhibition of mycelial growth during the experimental period was 22.0 (±1.5)%, the decreasing inhibitory trend toward the end of the assay indicates that CEO produced a transient delay in growth rather than sustained inhibition under these conditions. In contrast, *P. brevicompactum* showed a marked reduction in final diameter compared to the control, with a mean inhibition percentage of 51.3 (±1.9)% throughout the experiment, indicating sustained growth suppression under exposure to CEO. The greater sensitivity of this species may be related to differences in membrane composition and to its susceptibility to disruption by components of the terpene-rich essential oil. In addition, fungal membranes rely on ergosterol as the main sterol to maintain fluidity and structural integrity, and interference with ergosterol biosynthesis or direct interaction with membrane lipids by essential oil compounds can weaken the membrane and increase permeability, ultimately impairing fungal viability. These mechanisms have been documented for various essential oil components, including phenolic compounds that reduce ergosterol content and alter membrane function in various fungal species [37,38]. Furthermore, differences in growth kinetics or adaptive responses to stress could contribute to the species-specific behavior observed, as *P. brevicompactum* did not exhibit late-stage compensatory growth like *P. expansum*.

#### 2.1.3. Effect of CEO Treatment on Membrane Integrity

**Figure 3:**
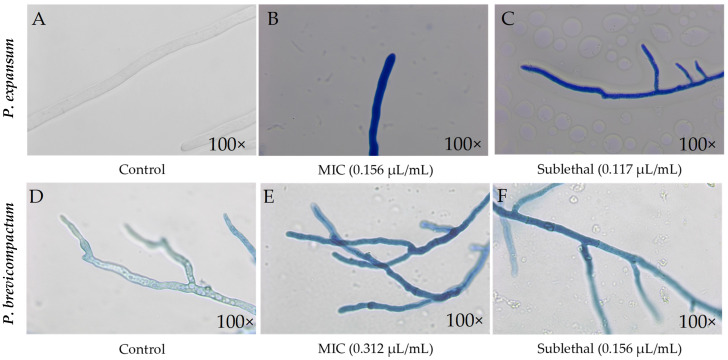
Effect on membrane integrity: (**A**,**D**), inoculated control on PDA agar; (**B**,**E**), MIC at a concentration of CEO; (**C**,**F**), sublethal concentration of CEO for each fungus. Evans blue staining indicates loss of membrane integrity, where blue-stained hyphae correspond to damaged cells, while unstained hyphae indicate intact membranes. Source: Castillo-Díaz et al., 2024 [31].

Both fungal species were exposed to predetermined concentrations of CEO, at both the MIC and a sublethal concentration, and subsequently stained with Evans blue to assess the state of their cell membranes. In both fungi, the treated samples showed significantly more intense and extensive staining than the untreated control, indicating a clear deterioration in membrane integrity after 2 h of exposure. Furthermore, no drastic morphological changes were observed. In the *P. expansum* control, the hyphae appeared healthy and showed no staining (Figure 3A), while those treated with the MIC showed deep staining (Figure 3B). The hyphae exposed to the sublethal dose showed complete staining and a thinner appearance (Figure 3C). In *P. brevicompactum*, regular hyphae with well-defined vacuoles were observed (Figure 3D). In contrast, in the treatments (Figure 3E,F), the vacuoles were not visible, and the hyphae appeared slightly thinner and septate.

The staining observed in the treated hyphae suggests a significant alteration of the fungal membrane, probably associated with the action of the essential oil components, particularly terpenes and terpenoids. These can interfere with fungal cell wall biosynthesis by affecting the transport of secretory vesicles that carry β-(1,3)-glucan synthase to the plasma membrane. This alteration reduces β-glucan at the hyphal tip, weakening cell wall integrity and hindering growth, thereby compromising structural stability [37,39]. In addition, several studies report that essential oils can interfere with sterol biosynthesis, particularly ergosterol, a key lipid for maintaining the osmotic and metabolic stability of the fungal cell [40]. The role of terpenoid phenols in membrane depolarization has also been described. According to Rao et al. (2010) [41], the delocalized electronic systems of these molecules facilitate the dissociation of protons from OH groups, allowing the passage of monovalent cations and dissipating the pH gradient. Ahmad et al. (2013) [42] proposed that these compounds may inhibit H^+^-ATPase, the enzyme responsible for maintaining the electrochemical proton gradient and regulating transmembrane pH [43]. Its inhibition leads to intracellular acidification and, ultimately, cell lysis, an effect that may be even greater when combined with azole compounds.

#### 2.1.4. Effect of Essential Oil on Spore Germination

**Figure 4:**
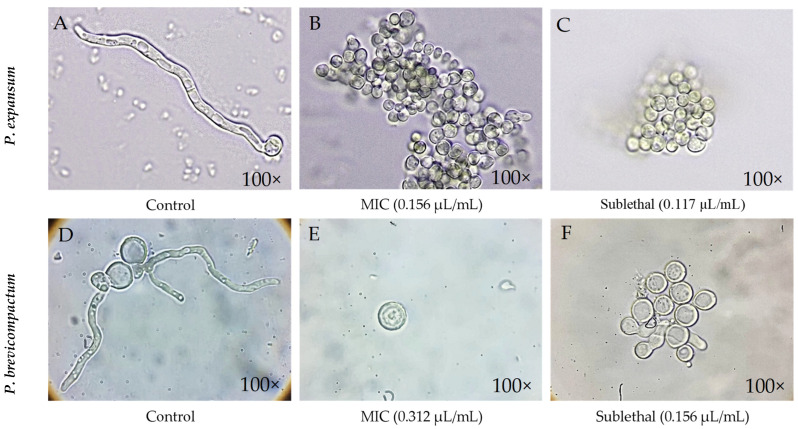
Effect on spore germination at 24 h: (**A**,**D**), inoculated control on PDA agar; (**B**,**E**), MIC at a concentration of CEO; (**C**,**F**), sublethal concentration of CEO for each fungus.

After counting the germinated spores in each treatment (MIC and sublethal concentration) and comparing them with their respective controls, it was observed that both treatments significantly reduced germination in both species after 24 h, as shown in Table 2. In the control samples (Figure 4A,D), no morphological changes were observed. In contrast, the treated spores showed considerably lower germination, without apparent or drastic morphological alterations in any case (Figure 4B,C,E,F). The values obtained indicate that the MIC and sublethal dose produce very similar effects in both *Penicillium* species, as germination percentages were similar and no significant differences were observed between treatments. The decrease in germination could be related to the combined action of the compounds present in the essential oil, whose synergistic effects may interfere with the initial processes of fungal growth [44]. This forces microorganisms to activate adaptation mechanisms to cope with chemical stress, delaying their ability to germinate and develop normally [14]. This behavior coincides with the increase in the lag phase observed in both species in the Gompertz model.

### 2.2. Identification of Volatile Compounds in Clove Essential Oil (S. aromaticum) by Gas Chromatography Coupled with Mass Spectrometry (GC-MS)

The composition of clove essential oil (CEO) was identified by GC-MS, comparing retention indices and molecular weights with those of the Adams [45] and NIST05 [46] libraries. A total of 15 compounds were detected, of which the five most abundant are listed in Table 3, including eugenol (76.5%), eugenyl acetate (17.8%), and trans-β-caryophyllene (4.0%), as well as small amounts of α-humulene and trans-β-caryophyllene oxide (0.5%). These compounds are known for a wide variety of biological activities, especially antimicrobial [47], antifungal [48], anticancer [49], anti-inflammatory [50] and antioxidant [51], which supports their potential as biocontrol agents.

Traces of esters, ketones, terpenes, phenols, and phenolic ethers were also identified. As reported in previous studies, the composition of essential oils can vary considerably due to intrinsic factors such as plant age, density, and genotype, as well as external factors including environmental conditions, geographical location, and extraction method [53]. These variations influence biosynthetic pathways and can give rise to different chemotypes or ecotypes, which, in turn, modify the oil’s biological activity, including its antimicrobial, antioxidant, and larvicidal properties [44].

### 2.3. In Silico Analysis

#### 2.3.1. ADMET Results for Components of Clove Essential Oil

The toxicological analysis performed using the ProTox 3.0 platform allowed us to estimate the toxicity level (Tox-Level) of the major compounds in CEO (Table 4). In general, the compounds evaluated were classified into toxicity categories 4 and 5 according to the GHS, corresponding to substances of moderate and low toxicity, respectively. This behavior is consistent with what has been previously reported for secondary metabolites derived from essential oils, which tend to have acceptable toxicological profiles due to their terpenoid nature and frequent use in natural formulations [50]. Among the compounds analyzed, UDP-GlcNAc, included as a reference biological ligand, was classified in category 6, corresponding to practically non-toxic substances. This was to be expected, given that it is an endogenous molecule widely involved in essential metabolic pathways and with no evidence of toxic effects at physiological concentrations. As for the components of the essential oil, eugenol, the main molecule in CEO with more than 70% relative abundance, had a Tox-Level of 4, corresponding to moderate toxicity. This result is consistent with previous studies that indicate that, although eugenol is safe in low doses, it can have cytotoxic or irritant effects at high concentrations [54]. Eugenyl, also classified in category 4, showed similar behavior, suggesting that the esterification of eugenol does not drastically reduce its toxicological profile. For their part, the sesquiterpenes trans-β-caryophyllene, α-humulene, and caryophyllene oxide had a Tox-Level of 5, placing them in the low-toxicity category. This result is particularly relevant because these compounds have been reported to have anti-inflammatory and antifungal properties, even though they represent a smaller percentage within the essential oil, and their lower toxicity could contribute to a safer overall profile for CEO [55].

#### 2.3.2. Selection and Modeling of CHS I Enzyme

**Figure 5:**
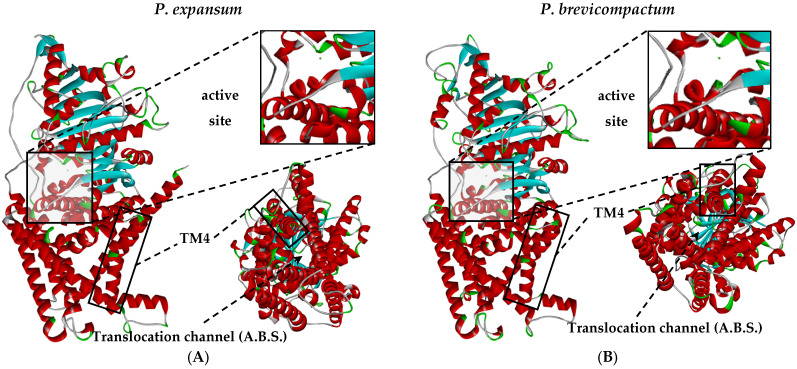
Three-dimensional model of the CHS I protein for *P. expansum* (**A**) and *P. brevicompactum* (**B**), highlighting the active site and chitin translocation channel alongside the TM4 alpha helix that regulates this channel.

Chitin is a fundamental component of the fungal cell wall; therefore, it is an attractive target for inhibiting phytopathogenic fungi. Its synthesis is mediated by the enzyme chitin synthase I (CHS I), a transmembrane protein that is part of a complex system responsible for chitin polymerization. Based on this, this computational study explored the possible interactions between the significant clove compounds, essential oil, and the CHS I enzymes of *P. brevicompactum* and *P. expansum*. In addition, possible conformational changes that could suggest an inhibition mechanism associated with these compounds were evaluated. The three-dimensional structures of the enzyme for each species were obtained from their primary sequences available at NCBI (XP_016602896.1 and KAJ5367747.1 for *P. expansum* and *P. brevicompactum*, respectively), using two current modeling platforms: AlphaFold2 and SWISS-MODEL. Figure 5A,B show the three-dimensional structures of the CHS I enzyme used in the analyses.

For *P. expansum* (Figure 5A), AlphaFold2 produced a structural model with an average pLDDT score of 81.12, which falls within the range interpreted as high-confidence. In AlphaFold2, pLDDT values above 90 indicate very high model reliability, whereas scores below 70 denote regions with low confidence in the predicted conformation. In addition to pLDDT, we examined the Predicted Aligned Error (PAE) profiles for all generated models to evaluate the reliability of domain positioning and residue-to-residue relationships. This combined evaluation ensured that the selected model displayed both local structural confidence and acceptable global accuracy [56], with high similarity to a homologous CHS I from *Penicillium oxalicum* [57]. In the case of *P. brevicompactum* (Figure 5B), SWISS-MODEL produced a model with a GMQE of 0.76 [58], indicating a reliable prediction, using as the main template a protein from *Aspergillus bombycis* belonging to the same phylum Ascomycota [59]. After model selection, we performed structural trimming to remove terminal regions that did not contribute meaningful information for docking or molecular-dynamics analyses. These segments, which are typically flexible and poorly structured, can introduce noise or interfere with docking accuracy. In the *P. expansum* model, residues 1–39 were removed. In the *P. brevicompactum* model, residues 1–120 and 911–1100 were excluded. All removed regions were located far from both the active site (AS) and the allosteric binding site (ABS), and their elimination did not affect any residues relevant to catalytic or regulatory function.

**Figure 6:**
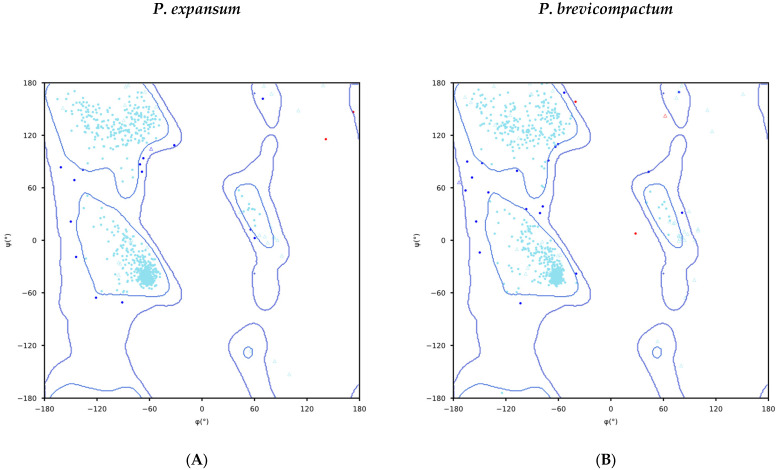
Ramachandran plots for *P. expansum* (**A**) and *P. brevicompactum* (**B**) show the φ/ψ dihedral angles of the main chain; dark blue and light blue contours indicate the most favored and additionally allowed regions, respectively, while red dots represent outliers.

To increase confidence in the selected structural models, we evaluated the stereochemical quality of each protein using Ramachandran plot statistics, which provide a measure of whether backbone dihedral angles fall within energetically plausible conformational regions (Figure 6). For the *P. expansum* CHS I model (Figure 6A), 98% of residues were located in favored regions, 1.8% in allowed regions, and fewer than 1% in disallowed regions. Only two residues, Ala725 and Asp727, fell into disallowed conformations, indicating localized deviations without compromising the global structure. Similarly, the *P. brevicompactum* model (Figure 6B) showed 96% of its residues in favored regions, 3% in allowed regions, and less than 1% in disallowed regions. In this case, the residues with non-ideal conformations were Pro134, Gly782, and Thr880. Overall, the high proportion of residues occupying favored conformational space, combined with the very small number of residues in disallowed regions, most of which are located in flexible or peripheral segments, supports the stereochemical soundness of both models. On this basis, the structures were considered sufficiently well-modeled for subsequent computational analyses [60,61,62].

Structural validation was performed by superimposing each model onto a crystallized CHS I from *Saccharomyces cerevisiae* (PDB 8K3W). The RMSD values obtained were 0.937 Å for *P. expansum* and 0.886 Å for *P. brevicompactum*, demonstrating high structural similarity and supporting the quality of the models. Furthermore, following previous reports [63], an Mg^2+^ ion was added to the active site of both enzymes, given its relevance as a cofactor in the catalytic activity of CHS I.

#### 2.3.3. Ligand Selection

**Figure 7:**
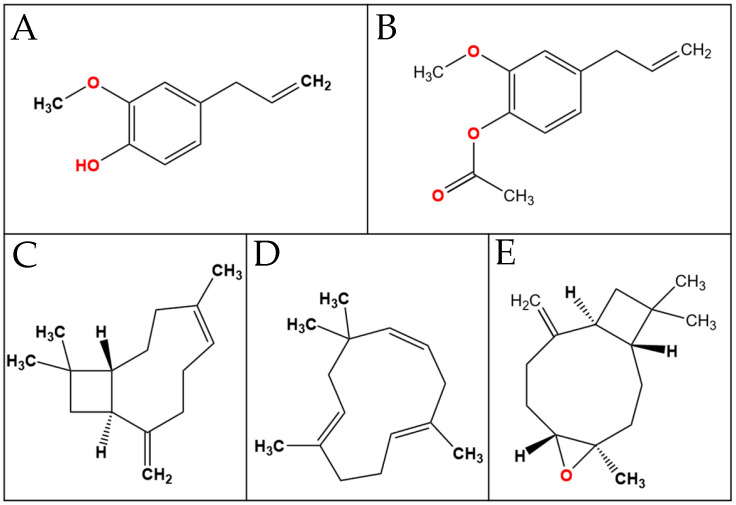
Main components of CEO: (**A**), eugenol; (**B**), eugenyl acetate; (**C**), trans-β-caryophyllene; (**D**), α-humulene; and (**E**), trans-β-caryophyllene oxide.

Ligand selection was based on the chemical profiles obtained from GC–MS CEO analysis and on preliminary ADMET property evaluations. Once the compounds present had been identified, the five primary metabolites were selected, considering that the most abundant molecules tend to contribute significantly to the biological activity of the essential oil. In addition, various studies indicate that a higher proportion of certain terpenes and phenylpropanoids may be directly associated with the observed antifungal activity, while the minor components may act synergistically to enhance this effect [64]. The selected compounds were eugenol, eugenyl acetate, trans-β-caryophyllene, α-humulene, and trans-β-caryophyllene oxide (Figure 7). These compounds showed favorable ADMET profiles, supporting their selection as candidates for computational studies. Subsequently, each ligand was optimized using Avogadro v1.2, Gaussian 09, Discovery Studio Visualizer v24, and AutoDock Tools to obtain stable, energetically favorable structures for molecular docking and molecular dynamics simulations.

#### 2.3.4. Molecular Docking Results

A molecular docking was performed for each of the selected ligands from CEO, evaluating their ability to interact with the CHS I enzymes of *P. expansum* and *P. brevicompactum*, both at the active site (AS) and at a proposed allosteric binding site (ABS).

The docking analysis provided a comparison of the potential interactions between the clove essential oil components and CHS I. These simulations are exploratory and help generate hypotheses about a possible enzyme inhibition mechanism influenced by the CEO components.

The identification of this ABS was based on an exploratory cavity analysis performed with CB-Dock 2, which revealed potentially accessible and energetically favorable regions for ligand binding beyond the classical catalytic site.

It should be noted that, unlike the AS, this allosteric site has not been extensively studied in CHS I of phytopathogenic fungi, despite its proximity to the transmembrane regions associated with the translocation of the newly synthesized chitin chain. In this context, the selection of this ABS is particularly relevant, since the structural modulation of these regions could interfere with the opening of the translocation channel and, therefore, with the correct export of the polymer to the cell wall. The binding energy values obtained are summarized in Table 5. As a reference, the UDP-GlcNAc molecule, a natural ligand of CHS I, evaluated only at the AS, was included in order to establish a direct comparison with the treatment compounds. In both fungi, the results showed that UDP-GlcNAc had the most favorable binding energy (−9.5 kcal/mol in *P. expansum* and −9.6 kcal/mol in *P. brevicompactum*), as expected given that the enzyme is structurally optimized to recognize this substrate. This affinity is explained by its ability to form hydrogen bonds and an Mg^2+^-stabilized ionic bond, interactions that are fundamental for the stabilization of the catalytic complex [65]. In addition, π-Sigma, π–alkyl, π–π stacked interactions, and salt bridges were identified, which provide additional stability to the complex [66,67].

**Figure 8:**
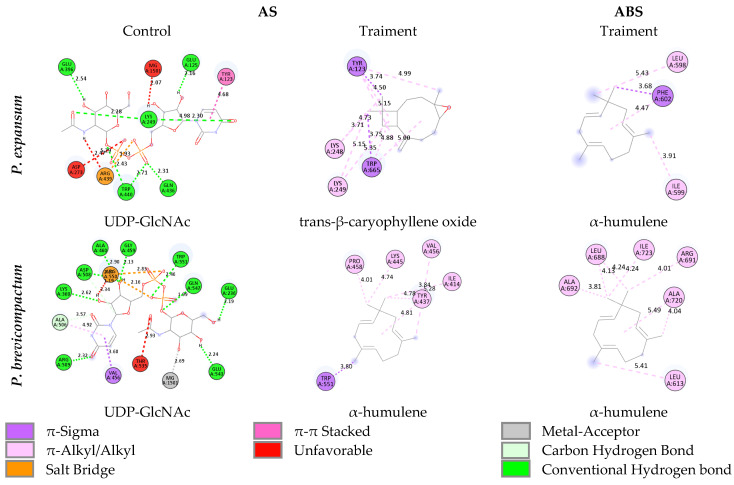
Comparison of interactions in ligand–receptor complexes (Control and Treatments—CHS I) for *P. expansum* and *P. brevicompactum* at the active site (AS) and allosteric binding site (ABS). Dashed lines represent intermolecular interactions between ligand and receptor, while colors indicate the type of interaction as defined by Discovery Studio Visualizer v24.

In docking studies, binding energies below −6.0 kcal/mol are generally considered biologically relevant, while values approaching −7.0 kcal/mol or lower suggest potential inhibitory activity through stable non-covalent interactions such as hydrophobic contacts and π–alkyl interactions [68]. This affinity, although not surpassing the natural substrate UDP-GlcNAc (−9.5 kcal/mol), is sufficiently high to imply a potential competitive binding at the catalytic site, which could interfere with chitin polymerization and enzyme function [29,63]. In addition, their predominantly lipophilic nature favored the formation of hydrophobic interactions, mainly π–sigma, π–alkyl, and alkyl, as shown in Figure 8. It is noteworthy that the CEO compounds share certain key residues with the natural ligand, which could indicate a possible competitive inhibition mechanism. In *P. expansum*, both UDP-GlcNAc and the treatment compounds interacted with TYR123 and LYS249, while in *P. brevicompactum,* they coincided at TRP551 and VAL456. These similarities suggest that the major metabolites of CEO may interfere with the recognition of the natural substrate [69].

On the other hand, to explore alternative mechanisms, coupling at a possible allosteric site was also evaluated. In this case, there is no natural reference ligand, so the comparison focused on the relative affinities between compounds. α-Humulene showed the most favorable values in both enzymes (−6.1 and −6.2 kcal/mol), followed by trans-β-caryophyllene oxide and trans-β-caryophyllene. In contrast, eugenol and eugenyl acetate showed lower affinities, likely due to the presence of polar functional groups that do not fit well with the site’s hydrophobic nature. The interactions in the ABS were mainly of the π–alkyl, alkyl, and π–Sigma type, established with hydrophobic residues such as PHE602, LEU598, and ILE599 in *P. expansum*, as well as ILE723, LEU688, ALA692, and ALA720 in *P. brevicompactum*. This pattern aligns with recent studies showing that enzyme–ligand complexes stabilized by hydrophobic interactions contribute to the antifungal activity of various bioactive compounds [70].

It is important to point out that although eugenol is a major constituent of the clove essential oil, its comparatively weaker docking scores against CHS I in both *Penicillium* models indicate that it is unlikely to act as a primary binder of this enzyme in our in silico framework. This should not be taken to mean that eugenol is irrelevant to the in vitro antifungal response. Rather, its contribution likely arises through complementary mechanisms, for example, membrane perturbation or indirect pathway effects, and/or through synergistic interactions with more hydrophobic minor constituents (α-humulene, trans-β-caryophyllene oxide) that show stronger predicted engagement with CHS I [39,71]. In this sense, the whole-oil activity observed in vitro could be interpreted as multimodal, with eugenol supporting membrane-level stress while minor sesquiterpenes provide potential target-level modulation, consistent with the established mixture behavior of essential oils.

#### 2.3.5. Molecular Dynamics Simulation Results

Molecular dynamics analysis was performed for 100 ns on the complexes that exhibited the highest bond energies during molecular coupling, both in AS and ABS, for *P. expansum* and *P. brevicosmpactum*, to compare their structural behavior.

It is important to explain that, for the ligand selection in molecular dynamics, score differences below ~1–2 kcal·mol^−1^ fall within the expected uncertainty of docking/scoring and are not interpreted as decisive rank separation [72,73]. Accordingly, we prioritize pose convergence, shared contacts with catalytic/TM residues, and site consistency (AS vs. ABS) over nominal rank [74]. Notably, the compounds we highlight (e.g., α-humulene, trans-β-caryophyllene oxide) achieve best docking scores at or beyond ~−7 kcal·mol^−1^, a practical screening threshold often used to flag plausible binders in the low-micromolar range by the relation ΔG = −RT ln Kd [68,74]. By contrast, eugenol scores are consistently weaker (≈−6 to −5 kcal·mol^−1^), aligning with its proposed contribution at the membrane level rather than via CHS I binding. Where relevant, we complement docking with MD-based analyses and plan replicate/consensus rescoring (e.g., short MD → MM/GBSA) to mitigate ranking noise [75,76].

In AS, three complexes were evaluated: a negative control (CHS I–UDP-GlcNAc), the treatment (CHS I–essential oil component), and the CHS I enzyme without ligands. In the case of ABS, two complexes were analyzed: CHS I with the essential oil component and CHS I without the ligand. All complexes were analyzed for their Root Mean Square Deviation (RMSD), Root Mean Square Fluctuation (RMSF), radius of gyration, and binding energy.

##### Root Mean Square Deviation (RMSD)

Root-mean-square deviation (RMSD) was used to evaluate the structural stability of CHS I enzymes and their protein–ligand complexes during molecular dynamics simulations. This parameter reflects global conformational changes over time and is widely used to assess the stability and persistence of interactions in molecular complexes [75]. In this study, the RMSD allowed us to compare the dynamic behavior of the complexes at the active site (AS) and at the allosteric binding site (ABS) of the modeled enzymes from *P. expansum* and *P. brevicompactum*.

**Figure 9:**
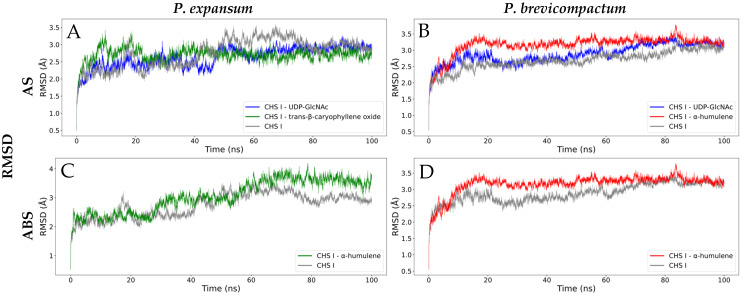

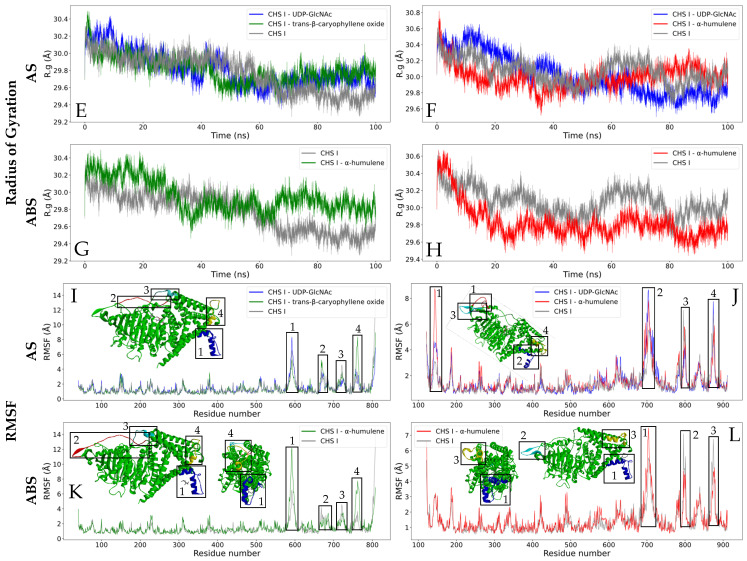
(**A**,**B**) RMSD graphs; (**E**,**F**) radius of gyration; (**I**,**J**) RMSF of the complexes formed between CHS I-UDP-GlcNAc, CHS I-CEO, and CHS I (alone) at the active site (AS) for *P. expansum* and *P. brevicompactum*. (**C**,**D**) RMSD graphs; (**G**,**H**) radius of gyration; (**K**,**L**) RMSF of complexes formed between CHS I-CEO and CHS I (alone) at the allosteric binding site (ABS) for *P. expansum* and *P. brevicompactum*.

For *P. expansum* in the AS (Figure 9A), the CHS I–trans-β-caryophyllene oxide complex exhibited the highest stability, characterized by initial fluctuations up to ~3.5 Å in the first 22 ns, followed by moderate variations (2.5–3.0 Å), suggesting consistent interaction attributed to the cyclic rigidity and hydrophobic nature of the ligand [77]. In contrast, CHS I–UDP-GlcNAc showed greater flexibility due to its multiple rotatable bonds, with more prolonged variations in the first 45 ns before stabilizing between 50 and 100 ns, a behavior consistent with its ability to establish numerous interactions with residues in the active site [78]. The enzyme in the apo state exhibited the greatest structural variability, consistent with the absence of restrictions generated by a ligand [79]. In *P. brevicompactum* (Figure 9B), a different pattern was observed: the enzyme in the apo state was the most stable, followed by the CHS I–UDP-GlcNAc complex and, finally, CHS I–α-humulene, whose highly hydrophobic nature could induce greater local perturbation and contribute to its overall instability [80]. These differences reflect variations in the residues that comprise the binding sites of the two modeled enzymes, which would explain the distinctive dynamic profiles observed.

In the possible ABS (Figure 9C,D), α-humulene led to a progressive increase in RMSD in both species, reaching up to 4.0 Å towards the end of the simulation. The apo protein showed minor fluctuations, suggesting that ligand binding could induce structural instability in this region [81]. This allosteric conformational alteration could be functionally relevant, since structural modifications distant from the AS can affect catalytic efficiency, supporting the hypothesis of an inhibitory mechanism mediated by an allosteric interaction.

##### Radius of Gyration (Rg)

The Rg is a fundamental parameter for evaluating the compaction and overall stability of a protein during a molecular dynamics simulation, as it describes the distribution of atoms relative to the center of mass and allows the identification of conformational expansion or contraction processes [82]. Sustained variations in Rg are often associated with relevant structural changes that can influence the functionality of the active site, while stabilization of Rg is interpreted as an indicator of the conformational integrity of the protein–ligand complex [83]. In *P. expansum*, the CHS I–UDP-GlcNAc and CHS I–trans-β-caryophyllene oxide complexes showed similar behavior in the AS (Figure 9E), starting with high Rg values and oscillations between 29.8 and 30.4 Å, followed by a progressive decrease to ~29.6 Å between 10 and 50 ns. This pattern suggests that ligand binding favors the compaction of the catalytic site, stabilizing the overall conformation of the enzyme, which is consistent with the RMSD results and with what has been previously reported for other enzymes [84]. The apo enzyme exhibited greater fluctuations, reflecting a more flexible conformation less restricted by the absence of a ligand. A comparable pattern was observed in *P. brevicompactum*, although with greater initial variability in the complexes, especially during the first 50 ns (Figure 9F). From this point on, the Rg values tended to stabilize and decrease slightly, again suggesting compaction induced by the presence of the ligand. The differences among the evaluated ligands appear to influence the degree of compaction achieved. At the same time, the apo state exhibited the greatest structural variability, reinforcing the relationship between ligand absence and increased conformational freedom.

In ABS, contrasting behaviors were recorded between the two species. In *P. expansum* (Figure 9G), the complex with the ligand had a higher Rg than the apo state, especially in the last 40 ns, indicating a conformational expansion induced by allosteric binding, consistent with the increase in RMSD and the greater overall flexibility of the complex [85]. In contrast, in *P. brevicompactum* (Figure 9H), the Rg of the complex was lower and more stable than that of the enzyme in the apo state, suggesting ligand-induced compaction at the allosteric site. This behavior could be related to the conformational adjustments necessary to maintain this compaction, which would explain the fluctuations observed in the RMSD.

##### Root Mean Square Fluctuation (RMSF)

Root-mean-square fluctuation (RMSF) allows the local flexibility of each residue during molecular dynamics to be evaluated and is key to identifying stable or mobile regions within a protein [86]. In protein–ligand complexes, this parameter helps elucidate conformational changes in the active site and potential allosteric areas, as low values are usually associated with stable interactions. In contrast, high values indicate flexible loops or regions involved in ligand recognition [87]. Thus, RMSF analysis complements the interpretation of global stability as measured by RMSD and allows characterization of how ligands modulate the enzyme’s structural plasticity.

In the case of CHS I, its dynamics are particularly relevant due to its dual enzymatic function: chitin synthesis at the active site and translocation of the newly formed polymer through the transmembrane channel regulated by the TM4 helix, as described by Chen et al. (2023) [63]. This helix acts as a gate, modulating channel opening and allowing chitin export to the cell wall.

In the AS of *P. expansum*, RMSF showed four regions with high mobility (Figure 9I), with fluctuations ranging from 6 to 8 Å. The first peak corresponds to a segment of the TM4 helix, consistently observed in all complexes, indicating an intrinsic protein movement that intensifies in the presence of ligands, especially with UDP-GlcNAc, followed by the apo enzyme and, to a lesser extent, the complex with trans-β-caryophyllene oxide. The remaining regions correspond mainly to loops or disordered areas of high mobility, as reported in other similar systems [88]. In *P. brevicompactum* (Figure 9J), similar fluctuation patterns were identified, with the additional appearance of a peak at residues 130–150 for the CHS I–α-humulene complex. This increase, close to 4 Å, coincides with the lower stability observed in the RMSD and may be due to the ligand’s hydrophobic nature or its unnatural character for the enzyme, which could induce local disorder or repulsive interactions [80]. The TM4 helix also showed marked fluctuations (residues 690–730), reinforcing its role as a region of intrinsic mobility associated with the translocation mechanism [63]. In ABS, *P. expansum* presented four peaks in regions equivalent to those of AS (Figure 9K), with the complex with α-humulene registering the most significant fluctuations in peaks 1 and 4, with differences of up to 6 Å from the apo state. These regions are again located on segments of TM4, consistent with the ligand’s proximity to the transmembrane channel in the initial docking. Given that this region contains low-polarity residues, hydrophobic and π–σ or π–alkyl interactions between α-humulene and TM4 could favor relevant conformational adjustments [89]. In contrast, peaks 2 and 3 were more pronounced in the apo enzyme, suggesting that the ligand may partially restrict the natural flexibility of these regions. For *P. brevicompactum*, the allosteric study revealed three prominent peaks (Figure 9L), with the most significant influence of α-humulene again evident in the area corresponding to TM4, with variations close to 2 Å. This behavior supports the hypothesis that the ligand can modulate the translocation channel, as observed in *P. expansum*.

##### Binding Energy

**Figure 10:**
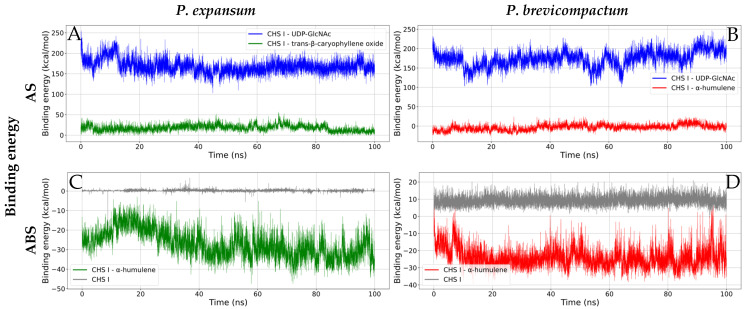
(**A**,**B**), binding energy graphs of the complexes formed between CHS I-UDP-GlcNAc and CHS I-CEO for *P. expansum* and *P. brevicompactum* at the active site (AS); (**C**,**D**), binding energy plots of the complexes formed between CHS I-CEO and CHS I (alone) for *P. expansum* and *P. brevicompactum* at the allosteric binding site (ABS).

The binding energy was calculated using the YASARA molecular dynamics module, whose macro uses an equation with an inverted sign compared to the traditional formulation implemented in programs such as GROMACS [90]. To maintain standard interpretation and allow comparison with previous work, the values were adjusted (inverted) when presenting them in the results, ensuring consistency with the conventional criteria used in protein–ligand studies. Based on the values reported in Table 6 and Figure 10, the natural substrate UDP-GlcNAc showed the highest affinities at the active site of both enzymes, with binding energies of 167.81 and 173.54 kcal/mol for *P. expansum* and *P. brevicompactum*, respectively. Although the positive values suggest reduced affinity on a direct scale, the presence of negative changes in potential energy (−201.97 and −158.65 kcal/mol) indicates the formation of stable non-covalent interactions during the simulation. However, the high solvation cost observed is consistent with the ligand’s polar and volumetric nature, which allows it to interact with solvent molecules at the catalytic site [68].

Among the CEO compounds evaluated at the active site, the binding energies were lower than those of the natural ligand. The CHS I–trans-β-caryophyllene oxide complex registered 17.56 kcal/mol, while CHS I–α-humulene registered −3.38 kcal/mol, suggesting greater stability for the latter during the simulation. Both complexes showed similar potential energy variations (−22.54 and −21.13 kcal/mol), indicating a comparable pattern of interactions due to the chemical similarity of the two sesquiterpenes. However, the difference in solvation energy (40.11 and 17.75 kcal/mol) reveals greater interaction of trans-β-caryophyllene oxide with the solvent, consistent with the presence of an epoxide group capable of forming contacts with water molecules, unlike α-humulene, which is more hydrophobic [68,91]. In the ABS, α-humulene showed considerably more favorable binding energies (−27.18 and −24.12 kcal/mol for *P. expansum* and *P. brevicompactum*, respectively). These results suggest that this compound would not compete directly with the natural substrate at the catalytic site but could act as an allosteric modulator or inhibitor. Its low polarity favors stable interactions in the enzyme’s transmembrane region, which is characterized by a predominance of hydrophobic residues. The negative potential energy values (−18.69 and −26.88 kcal/mol) support the stabilization of the complex through non-covalent interactions, while the reduced changes in solvation energy (−8.49 and 2.76 kcal/mol) reflect the low participation of the solvent, consistent with the hydrophobic nature of the ligand [91,92].

#### 2.3.6. Potential Allosteric Modulation

The computational results obtained in this study provide strong indications that clove essential oil (CEO) components may exert a dual inhibitory effect on chitin synthase I (CHS I) in *P. expansum* and *P. brevicompactum*. Docking analyses revealed that, although none of the CEO compounds surpassed the binding affinity of the natural substrate UDP-GlcNAc (−9.5 and −9.6 kcal/mol), several exhibited competitive energies at the active site (A.S.), notably trans-β-caryophyllene oxide (−7.9 kcal/mol) and α-humulene (−7.8 kcal/mol). These values fall within the range considered biologically relevant for enzyme inhibition [68,93], suggesting potential interference with substrate recognition.

Beyond orthosteric interactions, the evaluation of a proposed allosteric binding site (A.B.S.) uncovered additional inhibitory potential. α-Humulene displayed the most favorable binding energy at this site (−6.1 and −6.2 kcal/mol), forming predominantly hydrophobic interactions with residues such as PHE602, LEU598, and ILE599 in *P. expansum*, and ILE723, LEU688, and ALA692 in *P. brevicompactum*. These residues are located within the TM4 helix region, which is implicated in chitin translocation, a critical step in fungal cell wall assembly. The preference of sesquiterpenes for hydrophobic pockets distal to the catalytic core aligns with structural patterns reported for allosteric modulators in transmembrane enzymes [94,95].

Molecular dynamics (MD) simulations further support this hypothesis. RMSD and radius of gyration analyses demonstrated that ligand binding at the A.B.S. induced measurable conformational changes, including increased flexibility in TM4 segments and localized compaction in adjacent loops. RMSF profiles revealed dynamic perturbations in regions distant from the active site, consistent with allosteric regulation mechanisms described in the recent literature [63,96]. These findings suggest that CEO compounds may modulate CHS I activity not only by competing with UDP-GlcNAc at the catalytic site but also by altering structural dynamics essential for polymer translocation.

The integration of docking and MD data provides computational evidence for a putative allosteric mechanism of inhibition, in line with current paradigms emphasizing dynamic modulation over direct competition [94,95]. However, it is essential to note that while these results establish a robust theoretical framework, experimental validation, such as site-directed mutagenesis of A.B.S. residues or kinetic assays under varying ligand concentrations, is required to confirm the functional relevance of this mechanism.

## 3. Materials and Methods

### 3.1. Reagents

Clove essential oil (CEO, Marnys, Madrid, Spain), Tween 80 (Sigma-Aldrich, St. Louis, MO, USA), and potato dextrose agar (PDA; Sigma-Aldrich, MO, USA) were used to prepare the treatments. Evans blue staining (Sigma-Aldrich, St. Louis, MO, USA) was used for microscopic analysis.

### 3.2. Fungal Species

Two strains of phytopathogenic fungi were used in this study, *Penicillium expansum* DSMZ 1282 and *Penicillium brevicompactum* DSMZ 3825, sourced from the Faculty of Bioscience and Technology for Food, Agriculture, and the Environment at the University of Teramo, Italy.

### 3.3. In Vitro Study

#### 3.3.1. Antifungal Activity Assays

##### Effects on Mycelium Growth

The antifungal effect of the treatments was evaluated by measuring mycelial growth inhibition, as described by Peralta-Ruiz et al. (2020) [14]. Initially, an aqueous emulsion of clove essential oil was prepared with Tween 80 at 2% *v*/*v* relative to the essential oil concentration, and the emulsion was then incorporated into the PDA medium. Following this methodology, different concentrations of essential oil were formulated, starting at 1.25 µL/mL (*v*/*v*), and serial dilutions were performed until the minimum inhibitory concentration (MIC) and a sublethal concentration (below the MIC) were determined. For this, Petri dishes were inoculated with 5-mm-diameter discs obtained from independent cultures of *P. expansum* (4 days) and *P. brevicompactum* (5 days). Each treatment was performed using three independent biological replicates, with three technical replicates per biological replicate. The plates were incubated at 25 °C. Radial growth of the colonies was measured daily for 20 days in *P. expansum* and 17 days in *P. brevicompactum,* and subsequently analyzed with ImageJ v1.54g software [97]. The experimental data were fitted to the modified Gompertz equation (Equation (1)) proposed by Zwietering et al. (1990) [98] to estimate the growth parameters, *A*, *μ_m_* and *λ*.

(1) $$ln\left(\frac{D_{t}}{D_{0}}\right)=Ae^{{-e}^{\left[\frac{\mu_{m}e}{A}\left(\lambda -t\right)+1\right]}}$$

where

$D_{t}$ (cm): mean colony diameter at time *t*;
$D_{0}$ (cm): initial colony diameter;
A (cm): maximum growth reached in the stationary phase;
$\mu_{m}$ (cm/day): maximum radial growth rate;
λ (days): lag phase duration;
t (days): incubation time.

In addition, the percentage of mycelial growth inhibition was calculated using Equation (2), where *C* denotes the growth diameter of the control and *T* the diameter recorded in the presence of the treatment.

(2) Inhibition of mycelial growth (%)=(C−T)/C×100

It should be noted that colony diameter reflects radial expansion and does not directly quantify total biomass, hyphal density, or metabolic activity. Therefore, the inhibition values represent differences in surface growth rather than comprehensive fungal viability or physiological activity.

##### Effect of the CEO on Membrane Integrity

Damage to the fungal cell membrane was assessed by staining with Evans blue, following a protocol adapted from Chaves-Lopez et al. and Peralta-Ruiz et al. [14,99]. A total of 20 µL of a suspension of *P. expansum* and *P. brevicompactum* spores was inoculated onto slides covered with a thin layer of PDA to promote conidia germination. The slides were placed in Petri dishes conditioned as humidity chambers, sealed with parafilm, and incubated at 27 °C for 24 h. The germinated hyphae were then treated with an aqueous CEO emulsion for 2 h. After treatment, samples were washed with sterile physiological solution to remove residual reagents and stained with Evans blue for 5 min. Stained samples were examined under an optical microscope (B-290TB, Optika Science, Ponteranica, Italy) at 100× magnification. Three independent samples were analyzed per treatment. The evaluation was based on qualitative visual assessment of membrane integrity, and no quantitative image analysis or fluorescence measurements were performed.

##### Effect on Spore Germination

To standardize the spore suspensions, colonies of *P. expansum* and *P. brevicompactum* were grown on PDA for 5 and 16 days, respectively, until abundant sporulation was achieved. The spores were carefully collected using a sterile 0.9% NaCl solution to obtain a stock suspension. Serial dilutions were prepared, and OD_620_ values were measured to select suspensions close to 0.1 AU. Specific calibration curves were generated for each strain by correlating OD_620_ with the viable spore counts obtained after serial dilution and plating on PDA [14,100]. The spore concentration (spores/mL) was calculated using Equation (3):

(3) $$\frac{Spores}{mL}=\frac{number\;of\;colonies}{0.1\times dilution\;factor}$$

Then, the spore suspension was cultivated in microcentrifuge tubes containing potato dextrose broth and treated with MIC and a sublethal concentration of CEO, which were emulsified in the broth. After 24 h of incubation, a 20 µL aliquot was taken from each treatment and observed under an optical microscope (Compound microscope, Herwicm, China). In each sample, 100 spores were randomly counted and classified as germinated or non-germinated. Germination was determined by the presence of germ tubes longer than the conidia, a criterion defined by Peralta-Ruiz et al. (2020) [14]. All counts were performed by the same observer with identical optical settings to minimize inter-observer variability.

### 3.4. Identification of Volatile Compounds in Clove Essential Oil (S. aromaticum)

The composition of clove essential oil (CEO) was determined by gas chromatography–mass spectrometry (GC-MS) using the methodology described by Castro et al. (2024) [101]. The analysis was performed on an Agilent AT 6890 Series Plus gas chromatograph (Agilent Technologies, Palo Alto, CA, USA) coupled to a selective mass detector (MSD 5973N, Agilent Technologies), operating in full scan mode. Chromatographic separation was performed on a DB-5MS fused silica capillary column (J & W Scientific, Folsom, CA, USA; 60 m × 0.25 mm internal diameter × 0.25 μm film thickness), whose stationary phase consists of 5% phenyl and 95% dimethylpolysiloxane, suitable for resolving complex mixtures of volatile compounds. Helium was used as the carrier gas. The samples were diluted in dichloromethane and injected directly into the chromatograph using an injection volume of 2 μL in split mode (30:1). The mass spectrometer operated in electron ionization (EI) mode at 70 eV. A homologous series of hydrocarbons (C_6_–C_25_) was used as a reference to calculate retention indices. The final identification of each compound was confirmed by comparing the retention indices (RI) with the Adams (Wiley 138 database) [45] and the NIST Chemistry WebBook (NIST Standard Reference Database No. 69) [52].

### 3.5. In Silico Study

#### 3.5.1. ADMET Property Assessment

The ADMET (Absorption, Distribution, Metabolism, Excretion, and Toxicity) properties [102] of the selected compounds were evaluated in silico using the ProTox 3.0 platform https://tox-new.charite.de/protox_II/ (accessed on 15 March 2026). These analyses provided an initial approximation of the biological viability of the primary metabolites of clove essential oil. To this end, the chemical structures of each compound were obtained in SMILES format from the PubChem database. These SMILES codes were then manually entered into the ProTox interface using the server’s default parameters. This tool predicts properties related to toxicity and biological risk, including acute toxicity category, hepatotoxicity, carcinogenicity, mutagenicity, immunotoxicity, and other ADMET-related parameters [103]. The Globally Harmonized System (GHS) acute oral toxicity categories were interpreted as a continuous range from extremely toxic compounds (Category 1) to highly toxic (Category 2), toxic (Category 3), and harmful (Category 4), to those classified as low toxicity or cautionary (Category 5) [104]. Additionally, the practical category used by ProTox was considered, in which compounds with very low toxicity (equivalent to category 5–6; LD_50_ ≥ 2000 mg/kg) [103]. In this study, compounds classified in categories 5 to 6 were considered to have acceptably low toxicity, whereas those in categories 1 to 4 were considered to require caution or more detailed evaluation [91]. This categorization enabled the identification, in general terms, of the safety profile of each evaluated molecule.

It is important to note that the ProTox 3.0 outputs presented here refer to mammalian toxicity endpoints and GHS-style hazard categories that are trained and validated on rodent/human datasets; they are not direct measures of fungal toxicity or antifungal potency [103,104]. Accordingly, any statements regarding fungal safety or selectivity derived from ProTox should be understood as indirect and used only as early mammalian safety flags for individual constituents. Conclusions about antifungal activity in this work are instead based on our experimental MIC and germination assays, together with structure-guided hypotheses from docking and molecular dynamics against chitin synthase I, a fungal-specific target [27,105,106]. This division of roles, ADMET for mammalian hazard flagging and in vitro/in silico analyses for fungal efficacy and mechanism, is consistent with established practice in early discovery [107]. Moreover, because ergosterol and chitin biosynthetic pathways are absent in mammals, mammalian toxicity endpoints and fungal susceptibility probe distinct biological spaces [108].

#### 3.5.2. Selection and Modeling of the CHS I Protein

The protein chitin synthase I (CHS I), a member of the hexosyltransferase family within the GT2 glycosyltransferase group, was selected as the target molecule because of its critical role in chitin biosynthesis and translocation from the cytoplasm to the fungal cell wall. Chitin is an essential structural component of fungal cell walls, and CHS I catalyzes its polymerization and export, making this enzyme a strategic point for antifungal intervention.

In this study, we explicitly focused our computational analysis on chitin synthase I (CHS I; Class I) from *Penicillium expansum* and *P. brevicompactum*. Chitin synthases form a multigene family with distinct classes (I–VII) that play non-redundant roles in fungal growth and morphogenesis (Chs1/CHS I in wall repair, Chs2/CHS II in primary septum formation, and Chs3/CHS IV in bulk cell-wall chitin) [25,27]. We selected CHS I as the starting point for three practical reasons. First, recent structural work on Class I enzymes provides mechanistically anchored templates, including a well-defined catalytic core and the TM4-regulated translocation channel, that allow us to probe both orthosteric and TM-adjacent allosteric regions with greater confidence [63,105,106]. Second, the active-site architecture of CHS I is conserved across Ascomycota, supporting reliable homology/AI-assisted modeling for *Penicillium* and enabling straightforward mapping of catalytic residues and Mg^2+^ coordination (Ren et al., Brain et al.) [25,105]. Third, given the biology of *Penicillium* (rapid hyphal extension and chitin deposition), CHS I offers a relevant, structurally tractable target that links our whole-oil in vitro phenotypes to testable, target-level hypotheses in docking and MD (orthosteric competition vs. TM-proximal modulation). This focus does not exclude other CHS classes; rather, it provides a clear mechanistic entry point for a first-pass analysis, with CHS II/III/IV prioritized for future expansion as isoform-specific models and annotations continue to mature [25,27].

Although structural and in silico studies of chitin synthases have been reported in other organisms—such as the modeling of the catalytic region of *Chilo partellus* chitin synthase (CpCHS) for docking and molecular dynamics analyses with UDP-GlcNAc, nikkomycin, and polyoxin [109], as well as models of *Saccharomyces cerevisiae* and *Candida albicans* in different functional states [105,106] no studies have addressed the modeling or docking analysis of CHS I in species of the genus *Penicillium*. This gap adds novelty to the present work.

Since no crystal structure for CHS I from *P. expansum* or *P. brevicompactum* was available in the Protein Data Bank (PDB), the three-dimensional structures of both enzymes were generated by homology modeling using their primary sequences in FASTA format, retrieved from the National Center for Biotechnology Information (NCBI) https://www.ncbi.nlm.nih.gov/ (accessed on 15 March 2026). Structural prediction was performed with AlphaFold v2 https://github.com/sokrypton/ColabFold (accessed on 15 March 2026) [110] and SWISS-MODEL https://swissmodel.expasy.org/ (accessed on 15 March 2026) [111].

For each *Penicillium* species, we generated two preliminary CHS I structural models, one using AlphaFold/ColabFold and another using SWISS-MODEL. From these, we selected a single working model per species by applying a defined series of quality assessments focused on the structural regions most critical for function, followed by subsequent docking studies. For the AlphaFold-based models, our first criterion was local confidence across the GT2 catalytic core and the transmembrane (TM) helices. We evaluated both pLDDT values and Predicted Aligned Error (PAE) profiles, giving preference to models showing consistently higher confidence within these function-defining elements [56,110]. For the SWISS-MODEL structures, we next examined template-derived quality using GMQE scores, and we assessed overall stereochemical validity through Ramachandran analysis using RamPlot (available online: https://www.ramplot.in/) [62]. Only models displaying acceptable global geometry were retained for downstream docking and molecular-dynamics simulations [58,111,112].

We then compared all candidate structures with experimentally solved CHS homologs by aligning them to available yeast and *Candida* chitin-synthase structures. This step allowed us to verify the conservation of active-site geometry, the orientation of the TM4 “gate,” and the continuity of the predicted chitin-translocation channel [63,105,106]. Finally, we inspected each model for preservation of key sequence motifs and correct shaping of the catalytic pocket, including the positioning of the Mg^2+^-binding site reported in recent CHS structures [63,105]. This multi-step evaluation strategy follows established recommendations for integrating AI-based predictions with comparative modeling in studies of large membrane enzymes [113].

The resulting models were subsequently aligned in PyMOL 3.0.4 [114] against a crystallographic template available in the PDB (code 8K3W); https://www.rcsb.org/ (accessed on 15 March 2026) [63], corresponding to CHS I from *S. cerevisiae*, a phylogenetically related species within Ascomycota.

This approach follows the recommendations of Fiser [113], which emphasizes that using evolutionarily related templates enhances the reliability of comparative models. The alignment enabled evaluation of structural quality, identification of potential active sites, and verification of cofactor positions—features recognized as critical for improving functional interpretation of modeled structures, as highlighted by Hekkelman et al. (2023) [115]. This combined strategy yielded robust, biologically meaningful models, establishing a solid foundation for subsequent docking and molecular dynamics analyses.

To minimize artifacts from low-confidence, unstructured termini, the models were truncated to the last well-resolved residues prior to docking and MD. This approach is commonly used when AlphaFold confidence is low at the ends of large membrane enzymes, and those segments are remote from the binding pockets under study, because highly flexible tails can introduce non-physical fluctuations, spurious contacts under periodic boundary conditions, and grid artifacts in docking [56,112,113]. In our case, the N-/C-terminal segments that were truncated displayed low pLDDT/high PAE, lay > 20–30 Å from the orthosteric and putative TM-adjacent allosteric regions, and did not overlap the D,D,D/QxxRW catalytic motifs, the TM4 gate, or the translocation lumen mapped from cryo-EM references [63,105,106].

#### 3.5.3. Ligand Selection and Optimization

For the in silico analyses, we followed the general methodology described by Kundu et al. (2021) [116] and Sierra-Hernandez et al. (2025) [91], introducing specific adaptations to fit the objectives of this study. Based on the chemical profile obtained through GC–MS, the five most abundant compounds in clove essential oil were selected as ligands for molecular docking and dynamics simulations.

The chemical structures of these compounds were retrieved from the PubChem database https://pubchem.ncbi.nlm.nih.gov/ (accessed on 15 March 2026) in SDF format, using either compound names or CID identifiers. Additionally, the enzyme’s natural substrate, UDP-GlcNAc, was included as a reference ligand to assess comparative binding affinity and determine whether the selected metabolites could compete with the endogenous molecule.

Ligand optimization was performed in two stages. First, each compound underwent preliminary energy minimization in Avogadro v1.2 [117] using a genetic algorithm, and the resulting conformers were exported in PDB format. Subsequently, these structures were refined in Gaussian 09 (Linux) [118] through a geometry optimization based on density functional theory (DFT), applying the wB97X-D hybrid functional and the 6-31G(d,p) basis set, specified with the command opt wb97xd/6-31g(d,p). This procedure ensured that all ligands exhibited stable, energetically favorable geometries suitable for subsequent docking and molecular dynamics simulations.

#### 3.5.4. Preparation of Ligands and Proteins

The optimized ligand structures were imported into Biovia Discovery Studio Visualizer v24 [119], where polar hydrogens were added, and Gasteiger charges were assigned, ensuring adequate representation of electrostatic interactions for docking. In parallel, protein models were prepared in AutoDock Tools v1.5.7 [120], incorporating polar hydrogens and assigning Kollman charges, following recommendations for optimizing ligand-receptor interaction. Once the preparation was complete, both the ligands and the proteins were exported in pdbqt format, as required by AutoDock Vina [93] to perform molecular docking analyses.

#### 3.5.5. Molecular Docking

To analyze the interactions between the significant compounds of the CEO and the CHS I enzymes of *P. expansum* and *P. brevicompactum*, two structural regions of interest were considered: the active site (AS) and a putative allosteric binding site (ABS). The latter was identified from exploratory molecular docking analysis using CB-Dock 2 https://cadd.labshare.cn/cb-dock2/ (accessed on 15 March 2026), a tool that allows automatic detection of potential cavities in the protein, which made it possible to propose an alternative binding site that could be involved in chitin translocation [121]. The location of the active site was determined by structural alignment with a homologous CHS I whose crystal structure is available in the PDB (8K3W), which allowed the position of the catalytic center and the Mg^2+^ ions associated with enzymatic activity to be identified. Molecular docking was performed with AutoDock Vina [93]. The natural ligand of the enzyme (UDP-GlcNAc) was included as a control for the active site, while no reference ligand was used in the ABS, as it was approached as an exploratory analysis. The coordinates and dimensions of the docking boxes for both sites were defined by their structures and are presented in Table 7. The evaluation of the protein–ligand complexes was based on binding affinity values estimated by AutoDock Vina (kcal/mol), selecting the conformations with the lowest energies as the most favorable. Finally, the best poses obtained were inspected and analyzed visually with Biovia Discovery Studio Visualizer v24.

#### 3.5.6. Molecular Dynamics

Based on AutoDock Vina docking results, the protein–ligand complexes with the highest affinities were selected for stability evaluation via molecular dynamics simulations. For this purpose, YASARA Dynamics (version 32.12.24) [122] was used, employing the AMBER14 force field [123], which has been widely validated in studies of protein–ligand complex stability in areas such as biocatalysis, peptide inhibitor design, and carbohydrate analog análisis [124,125,126], supporting its use in this work. Each complex was placed in a cubic simulation box, leaving a 5 Å margin around the active site and an 8 Å margin around the allosteric site, and periodic boundary conditions were applied to recreate a continuous environment. The simulations were performed under physiological conditions: pH 7.4, 0.9% NaCl to neutralize the system, 298 K, and a water density of 0.997 g/mL. The software automatically adjusted the pressure and volume according to the water’s density. A 100 ns per-complex window was adopted as an initial, mechanism-oriented timescale to evaluate pose retention, local pocket rearrangements, and backbone/side-chain equilibration in the protein–ligand complexes. The reference and best-practice literature indicates that 10 to ~100 ns is typically sufficient to detect unstable docking poses, reach RMSD/Rg plateaus for the complex, as well as quantify residue-level flexibility (RMSF) for interpreting interaction patterns; while recognizing that slower, large-amplitude conformational events may require longer (μs-scale) trajectories [75,76]. In practice, numerous ligand-assessment studies on fungal or membrane-associated targets employ 50–100 ns as a pragmatic balance between convergence and computational cost, reporting stable discrimination of site-consistent binders within this window [24,83].

The md_analyze_dynamics.mcr macro was used to analyze the results, enabling the calculation of structural parameters, including root-mean-square deviation (RMSD), root-mean-square fluctuation per residue (RMSF), and radius of gyration (Rg) to assess the global and local stability of the complexes.

The bond energies were calculated taking into account the sign convention for these energies, and the YASARA macro md_analyzebindenergy.mcr was used, which defines the interaction energy as:

(4) $$∆E=E_{protein}+E_{ligand}-E_{Complex}$$

Under this convention, favorable interactions may yield positive values, which is opposite to the standard thermodynamic notation in which more negative energies indicate stronger binding. For consistency with the literature, the reported values were therefore expressed as:

(5) $${∆G}_{reported}=-∆E_{macro}$$

This adjustment does not modify the underlying physical calculation; it only harmonizes the macro’s outputs with the widely used biochemical sign convention. Similar sign corrections have been applied in prior studies using YASARA for protein–ligand binding analyses [122,124]. It is important to clarify that these values provide a comparative estimate for ranking poses within each system, not an absolute free-energy determination.

### 3.6. Statistical Analysis

For in vitro tests, differences between treatments and their respective controls were evaluated using Fisher’s LSD test to determine whether the variations observed were statistically significant. All analyses were performed using Statgraphics Centurion XVIII, with a confidence level of 95% (α = 0.05).

The graphical abstract was created using BioRender.com to illustrate the experimental workflow and key findings of this study.

## 4. Conclusions

This study provides comprehensive evidence that clove essential oil (CEO) is a promising natural antifungal agent against *P. expansum* and *P. brevicompactum*, combining strong experimental performance with mechanistic insights from computational modeling. In vitro assays demonstrated that CEO significantly inhibited mycelial growth and spore germination, achieving up to 85–90% growth reduction at 1.0% (*v*/*v*) and complete inhibition of spore germination at 0.5% (*v*/*v*). Membrane integrity tests revealed marked leakage of cellular contents, confirming severe structural disruption. These results underscore the practical potential of CEO for postharvest fungal control. In support of this, computational analyses revealed two complementary inhibitory mechanisms. Docking studies showed that sesquiterpenes such as trans-β-caryophyllene oxide (−7.9 kcal/mol) and α-humulene (−7.8 kcal/mol) exhibit strong affinity for the active site, interacting with key catalytic residues shared by UDP-GlcNAc. At the proposed allosteric site, α-humulene achieved binding energies of −6.1 to −6.2 kcal/mol, forming hydrophobic contacts with residues in the TM4 helix region. Molecular dynamics simulations confirmed these interactions, with RMSD stabilization after 20 ns, a reduction in radius of gyration from 30.4 Å to 29.6 Å, and reduced RMSFs in the TM4 segments, indicating conformational changes that could impair chitin translocation. These findings support a dual inhibitory mechanism: competitive interference at the catalytic site and a putative allosteric modulation of structural dynamics. This dual action not only enhances antifungal efficacy but also introduces a novel allosteric pocket in CHS I from *Penicillium* species, expanding the landscape for rational biocontrol design. While computational and experimental data converge to suggest functional relevance, future studies should validate these findings through mutagenesis and kinetic assays to optimize CEO stability and performance. Further studies will be addressed to determine if clove oil also suppresses chitin synthase gene expression, thereby revealing a complete multi-level antifungal mechanism. This integrated approach is essential to translate these promising results into an effective, sustainable postharvest solution.

## Figures and Tables

**Table 1:** Growth parameters for *P. expansum* and *P. brevicompactum* calculated using the modified Gompertz equation. A, Maximum diameter reached by the colony during the stationary phase (cm); µ_m_, maximum growth rate (cm/days); and λ, delay phase (days). Values are means ± standard deviation. Means followed by different letters in the same row are significantly different according to Fisher’s test (*p* < 0.05).

Parameters	*P. expansum*		*P. brevicompactum*	
	Control	Sublethal Concentration of CEO (0.117 µL/mL)	Control	Sublethal Concentration of CEO (0.156 µL/mL)
A (cm)	2.635 ± 0.003 a	2.733 ± 0.026 b	2.620 ± 0.036 a	2.484 ± 0.022 b
µm (cm/days)	0.355 ± 0.009 a	0.270 ± 0.021 b	0.309 ± 0.006 a	0.198 ± 0.006 b
λ (days)	0.361 ± 0.208 a	1.595 ± 0.126 b	0.142 ± 0.032 a	0.847 ± 0.133 b

**Table 2:** Effect of CEO treatments on the percentage of spore germination for *P. expansum* and *P. brevicompactum* after 24 h. Values are means ± standard deviation. Means followed by different letters in the same row are significantly different according to Fisher’s test (*p* < 0.05).

Fungal Species	% Spore Germination After 24 h		
*P. expansum*	Control	MIC (0.156 µL/mL)	Sublethal (0.117 µL/mL)
	96.0 ± 2.8 a	4.0 ± 1.4 b	4.5 ± 2.1 b
*P. brevicompactum*	Control	MIC (0.312 µL/mL)	Sublethal (0.156 µL/mL)
	87.5 ± 2.1 a	4.5 ± 6.4 b	5.5 ± 2.1 b

**Table 3:** Main volatile compounds identified in clove essential oil (*S. aromaticum*).

Chemical Group	Compound	Kovats Index		Retention Times (RT)	Relative Amount (%)
		Experimental	Literature		
Phenols	Eugenol	1362	1356 [45]	33.0	76.5
Esters	Eugenyl acetate	1519	1521 [45]	38.5	17.8
Sesquiterpenes	trans-β-caryophyllene	1432	1419 [52]	35.5	4.0
	α-humulene	1468	1454 [45]	36.8	0.5
sesquiterpenoids	trans-β-caryophyllene oxide	1596	1582 [45]	40.8	0.5

**Table 4:** ADMET results for major compounds in clove essential oil provided by ProTox.

Compound	Tox-Level	CID Pubchem
UDP-GlcNAc	6	445675
Eugenol	4	3314
Eugenyl acetate	4	7136
trans-β-caryophyllene	5	5281515
α-humulene	5	5281520
trans-β-caryophyllene oxide	5	1742210

**Table 5:** Binding energy scores in kcal/mol for each ligand–protein complex (compound of CEO and CHS I), for *P. expansum* and *P. brevicompactum* at the active site (AS) and allosteric binding site (ABS).

Compound	*P. expansum*		*P. brevicompactum*	
	AS	ABS	AS	ABS
UDP-GlcNAc	−9.5	-	−9.6	-
Eugenol	−6.5	−5.1	−6.0	−4.8
Eugenyl acetate	−6.5	−5.2	−6.5	−5.3
trans-β-caryophyllene	−7.8	−5.9	−7.4	−6.2
α-humulene	−7.7	−6.1	−7.8	−6.2
trans-β-caryophyllene oxide	−7.9	−5.9	−7.2	−6.1

**Table 6:** Average interaction energies of CHS I-ligand complexes, Δ potential energy, and Δ solvation energy for enzymes from each fungal species in both AS and ABS.

		Complex	Binding Energy (kcal/mol)	Δ Potential Energy (kcal/mol)	Δ Solvation Energy (kcal/mol)
*P. expansum*	AS	CHS I–UDP-GlcNAc	167.81	−201.97	369.78
		CHS I–trans-β-caryophyllene oxide	17.56	−22.54	40.11
	ABS	CHS I–α-humulene	−27.18	−18.69	−8.49
		CHS I (alone)	0.19	−0.06	0.26
*P. brevicompactum*	AS	CHS I–UDP-GlcNAc	173.54	−158.65	332.18
		CHS I–α-humulene	−3.38	−21.13	17.75
	ABS	CHS I–α-humulene	−24.12	−26.88	2.76
		CHS I (alone)	9.20	8.20	1.00

**Table 7:** Box parameters for molecular coupling execution.

Fungal Species and Active Site (AS) and Allosteric Binding Site (A.B.S) CHS I		Cartesian Coordinates of the Box			Box Size for Each Cartesian Coordinate (Å)		
		Center X	Center Y	Center Z	Size X	Size Y	Size Z
*P. expansum*	AS	184.771	156.582	157.016	30.75	36.75	30.75
	ABS	184.043	162.747	205.021	45.0	38.25	26.25
*P. brevicompactum*	AS	184.771	156.582	157.016	30.75	36.75	30.75
	ABS	184.043	162.747	205.021	45.0	38.25	26.25

## Data Availability

All data are available in the manuscript. The authors would like to clarify that some of the figures included in the present manuscript (Figure 2 and Figure 3) are based on preliminary results previously presented in a conference poster at the 5th International Electronic Conference on Foods (Castillo-Díaz et al., 2024) [31] and are properly cited.

## References

[1] Índice de Desperdicio de Alimentos 2021|UNEP—UN Environment Programme. https://www.unep.org/es/resources/informe/indice-de-desperdicio-de-alimentos-2021.

[2] Patiño B., Medina Á., Doménech M., González-Jaén M.T., Jiménez M., Vázquez C. (2007). Polymerase Chain Reaction (PCR) Identification of Penicillium Brevicompactum, a Grape Contaminant and Mycophenolic Acid Producer. Food Addit. Contam..

[3] Ndagijimana M., Chaves-López C., Corsetti A., Tofalo R., Sergi M., Paparella A., Guerzoni M.E., Suzzi G. (2008). Growth and Metabolites Production by Penicillium Brevicompactum in Yoghurt. Int. J. Food Microbiol..

[4] Overy D.P., Frisvad J.C. (2005). Mycotoxin Production and Postharvest Storage Rot of Ginger (Zingiber Officinale) by Penicillium Brevicompactum. J. Food Prot..

[5] Yu L., Qiao N., Zhao J., Zhang H., Tian F., Zhai Q., Chen W. (2020). Postharvest Control of Penicillium Expansum in Fruits: A Review. Food Biosci..

[6] Luciano-Rosario D., Keller N.P., Jurick W.M. (2020). Penicillium Expansum: Biology, Omics, and Management Tools for a Global Postharvest Pathogen Causing Blue Mould of Pome Fruit. Mol. Plant Pathol..

[7] Sharma A., Kumar V., Thukral A.K., Bhardwaj R. (2019). Responses of Plants to Pesticide Toxicity: An Overview. Planta Daninha.

[8] Alengebawy A., Abdelkhalek S.T., Qureshi S.R., Wang M.Q. (2021). Heavy Metals and Pesticides Toxicity in Agricultural Soil and Plants: Ecological Risks and Human Health Implications. Toxics.

[9] Mokhtarnejad L., Farzanehj M. (2024). The Effect of Thymol and Carvacrol Rich-Plant Essential Oils on Controlling Postharvest Decay Molds in Orange Fruit. Adv. Hortic. Sci..

[10] Da Silva A.M., Terao D., Vilela E.S.D., De Queiroz S.C.N., Maia A.H.N., Fracarolli J.A. (2025). Essential Oils on the Control of Postharvest Diseases of Papaya. An. Acad. Bras. Cienc..

[11] Kumar Pandey V., Shams R., Singh R., Dar A.H., Pandiselvam R., Rusu A.V., Trif M. (2022). A Comprehensive Review on Clove (*Caryophyllus aromaticus* L.) Essential Oil and Its Significance in the Formulation of Edible Coatings for Potential Food Applications. Front. Nutr..

[12] Giménez-Santamarina S., Torres-Pagan N., Larran S., Roselló J., Santamarina M.P. (2025). Clove Essential Oil and Eugenol as Natural Antifungal Agents to Reduce Postharvest Losses in Melon (*Cucumis melo*). Int. J. Mol. Sci..

[13] Yingprasert W., Matan N., Matan N. (2015). Effects of Surface Treatment with Cinnamon Oil and Clove Oil on Mold Resistance and Physical Properties of Rubberwood Particleboards. Eur. J. Wood Wood Prod..

[14] Peralta-Ruiz Y., Grande Tovar C., Sinning-Mangonez A., Bermont D., Pérez Cordero A., Paparella A., Chaves-López C. (2020). Colletotrichum Gloesporioides Inhibition Using Chitosan-Ruta Graveolens L Essential Oil Coatings: Studies In Vitro and In Situ on Carica Papaya Fruit. Int. J. Food Microbiol..

[15] Liu Q., Li L., Yang Z., Xiong X., Song Q., Li B., Zou H., Zhang L., Liu T. (2024). Antifungal Effect of Oregano Essential Oil Against Penicillium Expansum on Pyrus Sinkiangensis. J. Fungi.

[16] Ji Y., Hu W., Guan Y., Saren G. (2024). Effects of Plant Essential Oil Treatment on the Growth of Pathogenic Fungi and the Activity of Defense-Related Enzymes of Fungi-Inoculated Blueberry. Horticulturae.

[17] Mrvová M., Medo J., Lakatošová J., Barboráková Z., Golian M., Mašková Z., Tančinová D. (2024). Vapor-Phase Essential Oils as Antifungal Agents against Penicillium Olsonii Causing Postharvest Cherry Tomato Rot. Foods.

[18] Wang K., Ngea G.L.N., Godana E.A., Shi Y., Lanhuang B., Zhang X., Zhao L., Yang Q., Wang S., Zhang H. (2023). Recent Advances in Penicillium Expansum Infection Mechanisms and Current Methods in Controlling *P. expansum* in Postharvest Apples. Crit. Rev. Food Sci. Nutr..

[19] Magri A., Curci M., Battaglia V., Fiorentino A., Petriccione M. (2023). Essential Oils in Postharvest Treatment against Microbial Spoilage of the Rosaceae Family Fruits. AppliedChem.

[20] Lakshmayya N.S.V., Mishra A.K., Mohanta Y.K., Panda J., Naik B., Mishra B., Varma R.S. (2023). Essential Oils-Based Nano-Emulsion System for Food Safety and Preservation: Current Status and Future Prospects. Biocatal. Agric. Biotechnol..

[21] Sama-ae I., Pattaranggoon N.C., Tedasen A. (2023). In Silico Prediction of Antifungal Compounds from Natural Sources towards Lanosterol 14-Alpha Demethylase (CYP51) Using Molecular Docking and Molecular Dynamic Simulation. J. Mol. Graph. Model..

[22] Ahlawat V., Sura K., Singh B., Dangi M., Chhillar A.K. (2024). Bioinformatics Approaches in the Development of Antifungal Therapeutics and Vaccines. Curr. Genom..

[23] Yuan Y., Han R., Cao Q., Yu J., Mao J., Zhang T., Wang S., Niu Y., Liu D. (2017). Pharmacophore-Based Virtual Screening of Novel Inhibitors and Docking Analysis for CYP51A from Penicillium Italicum. Mar. Drugs.

[24] Ou-Ani O., Moujane S., Oucheikh L., Youssefi Y., Znini M., Chebabe D., Oubair A., Mabrouk E. (2023). Chemical Composition, In Vitro Antifungal Activity, Molecular Docking and Molecular Dynamics Simulation Studies of the Essential Oil of Ballota Hirsuta. J. Biol. Act. Prod. Nat..

[25] Brain L., Bleackley M., Doblin M.S., Anderson M. (2025). Fungal Chitin Synthases: Structure, Function, and Regulation. J. Fungi.

[26] Martínez J.P., Falomir M.P., Gozalbo D. (2014). Chitin: A Structural Biopolysaccharide with Multiple Applications. Encyclopedia of Life Sciences.

[27] Yang J., Zhang K.Q. (2019). Chitin Synthesis and Degradation in Fungi: Biology and Enzymes. Adv. Exp. Med. Biol..

[28] Ruiz-Herrera J., Manuel GonzÃ¡lez-Prieto J., Ruiz-Medrano R. (2002). Evolution and Phylogenetic Relationships of Chitin Synthases from Yeasts and Fungi. FEMS Yeast Res..

[29] Kulkarni S.A., Sellamuthu P.S., Anitha D.P.M., Madhavan T. (2021). In Vitro and in Silico Evaluation of Antifungal Activity of Cassia (*Cinnamomum cassia*) and Holy Basil (*Ocimum tenuiflorum*) Essential Oils for the Control of Anthracnose and Crown-Rot Postharvest Diseases of Banana Fruits. Chem. Pap..

[30] Bystrova E., Bogomolova E., Panina L., Bulianitsa A., Kurochkin V. (2008). Fungal Colony Patterning as an Example of Biological Self-Organization. Unifying Themes in Complex Systems IV.

[31] Castillo-Díaz Y.A., Grande-Tovar C.D., Castillo-Díaz Y.A., Grande-Tovar C.D. Essential Oil of Clavo (*Syzygium aromaticum*) as a Biocontrol Agent for the Phytopathogenic Fough Fungi Penicillium Brevicompactum and Penicillium Expansum. Proceedings of the 5th International Electronic Conference on Foods.

[32] Grande-Tovar C.D., Chaves-Lopez C., Viuda-Martos M., Serio A., Delgado-Ospina J., Perez-Alvarez J.A., Ospina N., la Tora S., Palmieri S., Paparella A. (2016). Sub-Lethal Concentrations of Colombian Austroeupatorium Inulifolium (H.B.K.) Essential Oil and Its Effect on Fungal Growth and the Production of Enzymes. Ind. Crops Prod..

[33] Robbins N., Caplan T., Cowen L.E. (2017). Molecular Evolution of Antifungal Drug Resistance. Annu. Rev. Microbiol..

[34] Berman J., Krysan D.J. (2020). Drug Resistance and Tolerance in Fungi. Nat. Rev. Microbiol..

[35] Gow N.A.R., Latge J.-P., Munro C.A. (2017). The Fungal Cell Wall: Structure, Biosynthesis, and Function. Microbiol. Spectr..

[36] Lee Y., Puumala E., Robbins N., Cowen L.E. (2020). Antifungal Drug Resistance: Molecular Mechanisms in Candida Albicans and Beyond. Chem. Rev..

[37] Leiva-Mora M., Bustillos D., Arteaga C., Hidalgo K., Guevara-Freire D., López-Hernández O., Saa L.R., Padilla P.S., Bustillos A. (2025). Antifungal Mechanisms of Plant Essential Oils: A Comprehensive Literature Review for Biofungicide Development. Agriculture.

[38] Tian F., Woo S.Y., Lee S.Y., Park S.B., Zheng Y., Chun H.S. (2022). Antifungal Activity of Essential Oil and Plant-Derived Natural Compounds against Aspergillus Flavus. Antibiotics.

[39] Zhao F., Li Q., Wu H., Huang J., Ju J. (2023). Synergistic Antifungal Mechanism of Effective Components from Essential Oil against Penicillium Roqueforti. Eng. Microbiol..

[40] Nazzaro F., Fratianni F., Coppola R., De Feo V. (2017). Essential Oils and Antifungal Activity. Pharmaceuticals.

[41] Rao A., Zhang Y., Muend S., Rao R. (2010). Mechanism of Antifungal Activity of Terpenoid Phenols Resembles Calcium Stress and Inhibition of the TOR Pathway. Antimicrob. Agents Chemother..

[42] Ahmad A., Khan A., Manzoor N. (2013). Reversal of Efflux Mediated Antifungal Resistance Underlies Synergistic Activity of Two Monoterpenes with Fluconazole. Eur. J. Pharm. Sci..

[43] Seto-Young D., Monk B., Mason A.B., Perlin D.S. (1997). Exploring an Antifungal Target in the Plasma Membrane H+-ATPase of Fungi. Biochim. Biophys. Acta (BBA)-Biomembr..

[44] Mena Palacios C., Silva López B., Medina A. (2020). Composición Química y Actividad Biológica de Los Aceites Esenciales de Lamiaceas, Asteraceas, Vervenaceas: Una Revisión. InfoANALÍTICA.

[45] Adams R.P. (2012). Identification of Essential Oil Components by Gas Chromatography/Mass Spectrometry.

[46] Linstrom P.J., Mallard W.G. (2001). The NIST Chemistry WebBook: A Chemical Data Resource on the Internet. J. Chem. Eng. Data.

[47] Tischer J.S., Possan H., Luiz J., Malagutti N.B., Martello R., Valério A., Dalmagro J., de Oliveira D., Oliveira J.V. (2019). Synthesis of Eugenyl Acetate through Heterogeneous Catalysis. J. Essent. Oil Res..

[48] Bezerra C.F., de Geraldo Alencar Júnior J., de Lima Honorato R., dos Lucas Santos A.T., da Pereira Silva J.C., da Gusmão Silva T., de Sampaio Freitas T., Fonseca Bezerra M.C., Alves Borges Leal A.L., Sales D.L. (2023). Antifungal Effect of the Liposome Encapsulation of the Trans- Caryophylene and Its Association with Fluconazole. Chem. Biol. Interact..

[49] Dahham S.S., Tabana Y.M., Iqbal M.A., Ahamed M.B.K., Ezzat M.O., Majid A.S.A., Majid A.M.S.A. (2015). The Anticancer, Antioxidant and Antimicrobial Properties of the Sesquiterpene β-Caryophyllene from the Essential Oil of Aquilaria Crassna. Molecules.

[50] Dalavaye N., Nicholas M., Pillai M., Erridge S., Sodergen M.H. (2024). The Clinical Translation of α-Humulene—A Scoping Review. Planta Med..

[51] Li W., Liu C., Deng J., Tang J., Jiang P., Ma L., Zeng X. (2017). Research on Properties of Polyvinyl Alcohol Antifungal Film Based on Clove Essential Oil Nano-Microcapsule. J. Comput. Theor. Nanosci..

[52] Linstrom P.J., Mallard W.G. (2026). NIST Chemistry WebBook, NIST Standard Reference Database No. 69.

[53] Mugao L. (2024). Factors Influencing Yield, Chemical Composition and Efficacy of Essential Oils. Int. J. Multidiscip. Res. Growth Eval..

[54] Said M.M., Rabo M.M.A. (2017). Neuroprotective Effects of Eugenol against Aluminiuminduced Toxicity in the Rat Brain. Arh. Hig. Rada Toksikol..

[55] Schmitt D., Levy R., Carroll B. (2016). Toxicological Evaluation of β-Caryophyllene Oil: Subchronic Toxicity in Rats. Int. J. Toxicol..

[56] Jumper J., Evans R., Pritzel A., Green T., Figurnov M., Ronneberger O., Tunyasuvunakool K., Bates R., Žídek A., Potapenko A. (2021). Highly Accurate Protein Structure Prediction with AlphaFold. Nature.

[57] Chitin Synthase-Penicillium Oxalicum (Strain 114-2/CGMCC 5302) (Penicillium Decumbens)|UniProtKB|UniProt. https://www.uniprot.org/uniprotkb/S8B6C7/entry.

[58] Biasini M., Bienert S., Waterhouse A., Arnold K., Studer G., Schmidt T., Kiefer F., Cassarino T.G., Bertoni M., Bordoli L. (2014). SWISS-MODEL: Modelling Protein Tertiary and Quaternary Structure Using Evolutionary Information. Nucleic Acids Res..

[59] NEDD8-Conjugating Enzyme UBC12-Aspergillus Bombycis|Genomic Coordinates|UniProtKB|UniProt. https://www.uniprot.org/uniprotkb/A0A1F8A0N4/entry#names_and_taxonomy.

[60] Balasco N., Esposito L., De Simone A., Vitagliano L. (2022). Local Backbone Geometry Plays a Critical Role in Determining Conformational Preferences of Amino Acid Residues in Proteins. Biomolecules.

[61] Park S.W., Lee B.H., Song S.H., Kim M.K. (2023). Revisiting the Ramachandran Plot Based on Statistical Analysis of Static and Dynamic Characteristics of Protein Structures. J. Struct. Biol..

[62] Kumar M., Rathore R.S. (2025). RamPlot: A Webserver to Draw 2D, 3D and Assorted Ramachandran (φ, ψ) Maps. J. Appl. Crystallogr..

[63] Chen D.D., Wang Z.B., Wang L.X., Zhao P., Yun C.H., Bai L. (2023). Structure, Catalysis, Chitin Transport, and Selective Inhibition of Chitin Synthase. Nat. Commun..

[64] Haro-González J.N., Barbosa-Nuñez J.A., Castillo-Herrera G.A., Estarrón-Espinosa M., Herrera-Rodríguez S.E., Espinosa-Andrews H., Álvarez Á.H., Martínez-Velázquez M. (2024). Clove Essential Oil and Its Major Component, Eugenol: A Comparative Study of Their in Vitro Antioxidant and Anticancer Properties. Nat. Prod. Res..

[65] Prejanò M., Alberto M.E., Russo N., Toscano M., Marino T. (2020). The Effects of the Metal Ion Substitution into the Active Site of Metalloenzymes: A Theoretical Insight on Some Selected Cases. Catalysts.

[66] Carter-Fenk K., Liu M., Pujal L., Loipersberger M., Tsanai M., Vernon R.M., Forman-Kay J.D., Head-Gordon M., Heidar-Zadeh F., Head-Gordon T. (2023). The Energetic Origins of Pi-Pi Contacts in Proteins. J. Am. Chem. Soc..

[67] Donald J.E., Kulp D.W., DeGrado W.F. (2011). Salt Bridges: Geometrically Specific, Designable Interactions. Proteins Struct. Funct. Bioinform..

[68] Du X., Li Y., Xia Y.L., Ai S.M., Liang J., Sang P., Ji X.L., Liu S.Q. (2016). Insights into Protein–Ligand Interactions: Mechanisms, Models, and Methods. Int. J. Mol. Sci..

[69] Abhishek S., Deeksha W., Nethravathi K.R., Davari M.D., Rajakumara E. (2023). Allosteric Crosstalk in Modular Proteins: Function Fine-Tuning and Drug Design. Comput. Struct. Biotechnol. J..

[70] Zhang Y.H., Yang S.S., Zhang Q., Zhang T.T., Zhang T.Y., Zhou B.H., Zhou L. (2023). Discovery of N-Phenylpropiolamide as a Novel Succinate Dehydrogenase Inhibitor Scaffold with Broad-Spectrum Antifungal Activity on Phytopathogenic Fungi. J. Agric. Food Chem..

[71] Ru Q., Huang Y., Jin Z., Yu D., Fu Z., Tian J., Guo M., Li X., Yang M., Luo J. (2025). Antifungal Activity and Inhibitory Mechanism of Essential Oil from Fresh and Fallen Leaves of *Cinnamomum camphora* (L.) Presl against Zearalenone-Producing Fusarium Graminearum. LWT.

[72] Warren G.L., Andrews C.W., Capelli A.M., Clarke B., LaLonde J., Lambert M.H., Lindvall M., Nevins N., Semus S.F., Senger S. (2005). A Critical Assessment of Docking Programs and Scoring Functions. J. Med. Chem..

[73] Li Y., Han L., Liu Z., Wang R. (2014). Comparative Assessment of Scoring Functions on an Updated Benchmark: 2. Evaluation Methods and General Results. J. Chem. Inf. Model..

[74] Zhao X., Liu Y., Liu X., Jiang J. (2018). Comparative Transcriptome Profiling of Two Tomato Genotypes in Response to Potassium-Deficiency Stress. Int. J. Mol. Sci..

[75] Guterres H., Im W. (2020). Improving Protein-Ligand Docking Results with High-Throughput Molecular Dynamics Simulations. J. Chem. Inf. Model..

[76] Genheden S., Ryde U. (2015). The MM/PBSA and MM/GBSA Methods to Estimate Ligand-Binding Affinities. Expert Opin. Drug Discov..

[77] Muhmood M.A., Safi F., Ahmed M.M., Almeani S.A.L. (2025). Syzygium Aromaticum Phytoconstituents Target SARS-CoV-2: Integrating Molecular Docking, Dynamics, Pharmacokinetics, and MiR-21 Rs1292037 Genotyping. Viruses.

[78] Wankowicz S.A., de Oliveira S.H.P., Hogan D.W., van den Bedem H., Fraser J.S. (2022). Ligand Binding Remodels Protein Side-Chain Conformational Heterogeneity. eLife.

[79] Stadler A.M., Pellegrini E., Johnson M., Fitter J., Zaccai G. (2012). Dynamics-Stability Relationships in Apo- and Holomyoglobin: A Combined Neutron Scattering and Molecular Dynamics Simulations Study. Biophys. J..

[80] Fusani L., Palmer D.S., Somers D.O., Wall I.D. (2020). Exploring Ligand Stability in Protein Crystal Structures Using Binding Pose Metadynamics. J. Chem. Inf. Model..

[81] Pan Y., Mader M.M. (2022). Principles of Kinase Allosteric Inhibition and Pocket Validation. J. Med. Chem..

[82] Lobanov M.Y., Bogatyreva N.S., Galzitskaya O.V. (2008). Radius of Gyration as an Indicator of Protein Structure Compactness. Mol. Biol..

[83] Chandra Babu T.M., Rajesh S.S., Bhaskar B.V., Devi S., Rammohan A., Sivaraman T., Rajendra W. (2017). Molecular Docking, Molecular Dynamics Simulation, Biological Evaluation and 2D QSAR Analysis of Flavonoids from Syzygium Alternifolium as Potent Anti-Helicobacter Pylori Agents. RSC Adv..

[84] Li H., Cao Z., Hu G., Zhao L., Wang C., Wang J. (2021). Ligand-Induced Structural Changes Analysis of Ribose-Binding Protein as Studied by Molecular Dynamics Simulations. Technol. Health Care.

[85] Oluyemi W.M., Samuel B.B., Adewumi A.T., Adekunle Y.A., Soliman M.E.S., Krenn L. (2022). An Allosteric Inhibitory Potential of Triterpenes from Combretum Racemosum on the Structural and Functional Dynamics of Plasmodium Falciparum Lactate Dehydrogenase Binding Landscape. Chem. Biodivers..

[86] Li D.D., Wu T.T., Yu P., Wang Z.Z., Xiao W., Jiang Y., Zhao L.G. (2020). Molecular Dynamics Analysis of Binding Sites of Epidermal Growth Factor Receptor Kinase Inhibitors. ACS Omega.

[87] Espíndola C. (2025). Modeling and Molecular Dynamics Studies of Flavone―DENV E-3 Protein―SWCNT Interaction at the Flavonoid Binding Sites. Viruses.

[88] Tobi D., Bahar I. (2005). Structural Changes Involved in Protein Binding Correlate with Intrinsic Motions of Proteins in the Unbound State. Proc. Natl. Acad. Sci. USA.

[89] Yao X.Q., Hamelberg D. (2024). Dissecting the Allosteric Fine-Tuning of Enzyme Catalysis. JACS Au.

[90] Abraham M.J., Murtola T., Schulz R., Páll S., Smith J.C., Hess B., Lindah E. (2015). GROMACS: High Performance Molecular Simulations through Multi-Level Parallelism from Laptops to Supercomputers. SoftwareX.

[91] Sierra-Hernandez O., Saurith-Coronell O., Rodríguez-Macías J., Márquez E., Mora J.R., Paz J.L., Flores-Sumoza M., Mendoza-Mendoza A., Flores-Morales V., Marrero-Ponce Y. (2025). In Silico Identification of Potential Clovibactin-like Antibiotics Binding to Unique Cell Wall Precursors in Diverse Gram-Positive Bacterial Strains. Int. J. Mol. Sci..

[92] Wu X., Guo Q., Li Q., Wan S., Li Z., Zhang J. (2022). Molecular Mechanism Study of EGFR Allosteric Inhibitors Using Molecular Dynamics Simulations and Free Energy Calculations. J. Biomol. Struct. Dyn..

[93] Trott O., Olson A.J. (2010). AutoDock Vina: Improving the Speed and Accuracy of Docking with a New Scoring Function, Efficient Optimization, and Multithreading. J. Comput. Chem..

[94] Zhu R., Wu C., Zha J., Lu S., Zhang J. (2025). Decoding Allosteric Landscapes: Computational Methodologies for Enzyme Modulation and Drug Discovery. RSC Chem. Biol..

[95] Lin A., Zhang Z., Jiang A., Li K., Shi Y., Yang H., Zhang J., Liu R., Wang Y., Glaviano A. (2025). Computational Approaches to Druggable Site Identification: Current Status and Future Perspective. Acta Pharm. Sin. B.

[96] Byun J.A., VanSchouwen B., Akimoto M., Melacini G. (2020). Allosteric Inhibition Explained through Conformational Ensembles Sampling Distinct “Mixed” States. Comput. Struct. Biotechnol. J..

[97] Schneider C.A., Rasband W.S., Eliceiri K.W. (2012). NIH Image to ImageJ: 25 Years of Image Analysis. Nat. Methods.

[98] Zwietering M.H., Jongenburger I., Rombouts F.M., Van’t Riet K. (1990). Modeling of the Bacterial Growth Curve. Appl. Environ. Microbiol..

[99] Chaves-Lopez C., Nguyen H.N., Oliveira R.C., Nadres E.T., Paparella A., Rodrigues D.F. (2018). A Morphological, Enzymatic and Metabolic Approach to Elucidate Apoptotic-like Cell Death in Fungi Exposed to h- and α-Molybdenum Trioxide Nanoparticles. Nanoscale.

[100] Oghaz N.A., Hatamzadeh S., Rahnama K., Moghaddam M.K., Vaziee S., Tazik Z. (2022). Adjustment and Quantification of UV-Visible Spectrophotometry Analysis: An Accurate and Rapid Method for Estimating *Cladosporium* spp. Spore Concentration in a Water Suspension. World J. Microbiol. Biotechnol..

[101] Castro J.I., Felipe A., Navas N., Valencia-Llano C.-H., Tenorio D.L., Grande-Tovar C.D., Castro J., Niebles A., Valencia-Llano C.-H., Lopez D. (2024). Polylactic Acid and Polycaprolactone Nanocomposites for Subdermal Tissue Regeneration. Prospectiva.

[102] Zhong H.A. (2017). ADMET Properties: Overview and Current Topics. Drug Design: Principles and Applications.

[103] Banerjee P., Kemmler E., Dunkel M., Preissner R. (2024). ProTox 3.0: A Webserver for the Prediction of Toxicity of Chemicals. Nucleic Acids Res..

[104] The United Nations Economic Commission for Europe (2023). Globally Harmonized System of Classification and Labelling of Chemicals (GHS): Tenth Revised Edition.

[105] Ren Z., Chhetri A., Guan Z., Suo Y., Yokoyama K., Lee S.Y. (2022). Structural Basis for Inhibition and Regulation of a Chitin Synthase from Candida Albicans. Nat. Struct. Mol. Biol..

[106] Wu Y., Zhang M., Yang Y., Ding X., Yang P., Huang K., Hu X., Zhang M., Liu X., Yu H. (2022). Structures and Mechanism of Chitin Synthase and Its Inhibition by Antifungal Drug Nikkomycin Z. Cell Discov..

[107] Bender A., Cortes-Ciriano I. (2021). Artificial Intelligence in Drug Discovery: What Is Realistic, What Are Illusions? Part 2: A Discussion of Chemical and Biological Data. Drug Discov. Today.

[108] Langjae R., Bussarawit S., Yuenyongsawad S., Ingkaninan K., Plubrukarn A. (2007). Acetylcholinesterase-Inhibiting Steroidal Alkaloid from the Sponge *Corticium* sp. Steroids.

[109] Ranganathan S., Ampasala D.R., Palaka B.K., Ilavarasi A.V., Patidar I., Poovadan L.P., Sapam T.D. (2020). In Silico Binding Profile Analysis and In Vitro Investigation on Chitin Synthase Substrate and Inhibitors from Maize Stem Borer, Chilo Partellus. Curr. Comput. Aided Drug Des..

[110] Mirdita M., Schütze K., Moriwaki Y., Heo L., Ovchinnikov S., Steinegger M. (2022). ColabFold: Making Protein Folding Accessible to All. Nat. Methods.

[111] Schwede T., Kopp J., Guex N., Peitsch M.C. (2003). SWISS-MODEL: An Automated Protein Homology-Modeling Server. Nucleic Acids Res..

[112] Afonine P.V., Poon B.K., Read R.J., Sobolev O.V., Terwilliger T.C., Urzhumtsev A., Adams P.D. (2018). Real-Space Refinement in PHENIX for Cryo-EM and Crystallography. Biol. Crystallogr..

[113] Fiser A. (2010). Template-Based Protein Structure Modeling. Methods Mol. Biol..

[114] Schrödinger LLC (2015). The PyMOL Molecular Graphics System.

[115] Hekkelman M.L., de Vries I., Joosten R.P., Perrakis A. (2023). AlphaFill: Enriching AlphaFold Models with Ligands and Cofactors. Nat. Methods.

[116] Kundu A., Dutta A., Mandal A., Negi L., Malik M., Puramchatwad R., Antil J., Singh A., Rao U., Saha S. (2021). A Comprehensive in Vitro and in Silico Analysis of Nematicidal Action of Essential Oils. Front. Plant Sci..

[117] Hanwell M.D., Curtis D.E., Lonie D.C., Vandermeerschd T., Zurek E., Hutchison G.R. (2012). Avogadro: An Advanced Semantic Chemical Editor, Visualization, and Analysis Platform. J. Cheminform..

[118] Carpenter J.E., Weinhold F. (1988). Analysis of the Geometry of the Hydroxymethyl Radical by the “Different Hybrids for Different Spins” Natural Bond Orbital Procedure. J. Mol. Struct. THEOCHEM.

[119] BIOVIA BIOVIA Discovery Studio Visualizer|Dassault Systèmes. https://www.3ds.com/products/biovia/discovery-studio/visualization.

[120] Morris G.M., Huey R., Lindstrom W., Sanner M.F., Belew R.K., Goodsell D.S., Olson A.J. (2009). AutoDock4 and AutoDockTools4: Automated Docking with Selective Receptor Flexibility. J. Comput. Chem..

[121] Liu Y., Yang X., Gan J., Chen S., Xiao Z.X., Cao Y. (2022). CB-Dock2: Improved Protein-Ligand Blind Docking by Integrating Cavity Detection, Docking and Homologous Template Fitting. Nucleic Acids Res..

[122] Krieger E., Vriend G. (2015). New Ways to Boost Molecular Dynamics Simulations. J. Comput. Chem..

[123] Dickson C.J., Madej B.D., Skjevik Å.A., Betz R.M., Teigen K., Gould I.R., Walker R.C. (2014). Lipid14: The Amber Lipid Force Field. J. Chem. Theory Comput..

[124] Land H., Humble M.S. (2018). YASARA: A Tool to Obtain Structural Guidance in Biocatalytic Investigations. Methods Mol. Biol..

[125] Prasasty V.D., Istyastono E.P. (2020). Structure-Based Design and Molecular Dynamics Simulations of Pentapeptide AEYTR as a Potential Acetylcholinesterase Inhibitor. Indones. J. Chem..

[126] Amin M.R., Yasmin F., Hosen M.A., Dey S., Mahmud S., Saleh M.A., Bin Emran T., Hasan I., Fujii Y., Yamada M. (2021). Synthesis, Antimicrobial, Anticancer, PASS, Molecular Docking, Molecular Dynamic Simulations & Pharmacokinetic Predictions of Some Methyl β-D-Galactopyranoside Analogs. Molecules.

